# Supplementary material for: Synthesis, Crystal Structure, and Biological Evaluation of Novel 5-Hydroxymethylpyrimidines
Source: Materials (Basel). 2021 Nov 16;14(22):6916. doi: 10.3390/ma14226916 (PMC8618934; doi:10.3390/ma14226916)
Supplement: Supplementary file 1 [file materials-14-06916-s001.zip › materials-1389384-supplementary.pdf]

materials

Article

Synthesis, Crystal Structure, and Biological Evaluation of Novel 5-Hydroxymethylpyrimidines

Marcin Stolarczyk [1], Agnieszka Matera-Witkiewicz [2,*], Aleksandra Wolska [2], Magdalena Krupińska[2], Aleksandra Mikołajczyk [2], Anna Pyra [3] and Iwona Bryndal [1]

1. Department of Organic Chemistry and Drug Technology, Faculty of Pharmacy, Wroclaw Medical University, 211A Borowska, 50-556 Wroclaw, Poland; marcin.stolarczyk@umw.edu.pl (M.S.); iwona.bryndal@umw.edu.pl (I.B.)
2. Screening Laboratory of Biological Activity Test and Collection of Biological Material, Faculty of Pharmacy, Wroclaw Medical University, 211A Borowska, 50-556 Wroclaw, Poland; aleksandra.wolska@umw.edu.pl (A.W.); magdalena.krupinska@umw.edu.pl (M.K.); aleksandra.mikolajczyk@umw.edu.pl (A.M.)
3. Faculty of Chemistry, University of Wroclaw, Joliot-Curie Street 14, 50-383 Wroclaw, Poland; anna.pyra@chem.uni.wroc.pl

* Correspondence: agnieszka.matera-witkiewicz@umw.edu.pl; Tel.: +48-71-784-06-68

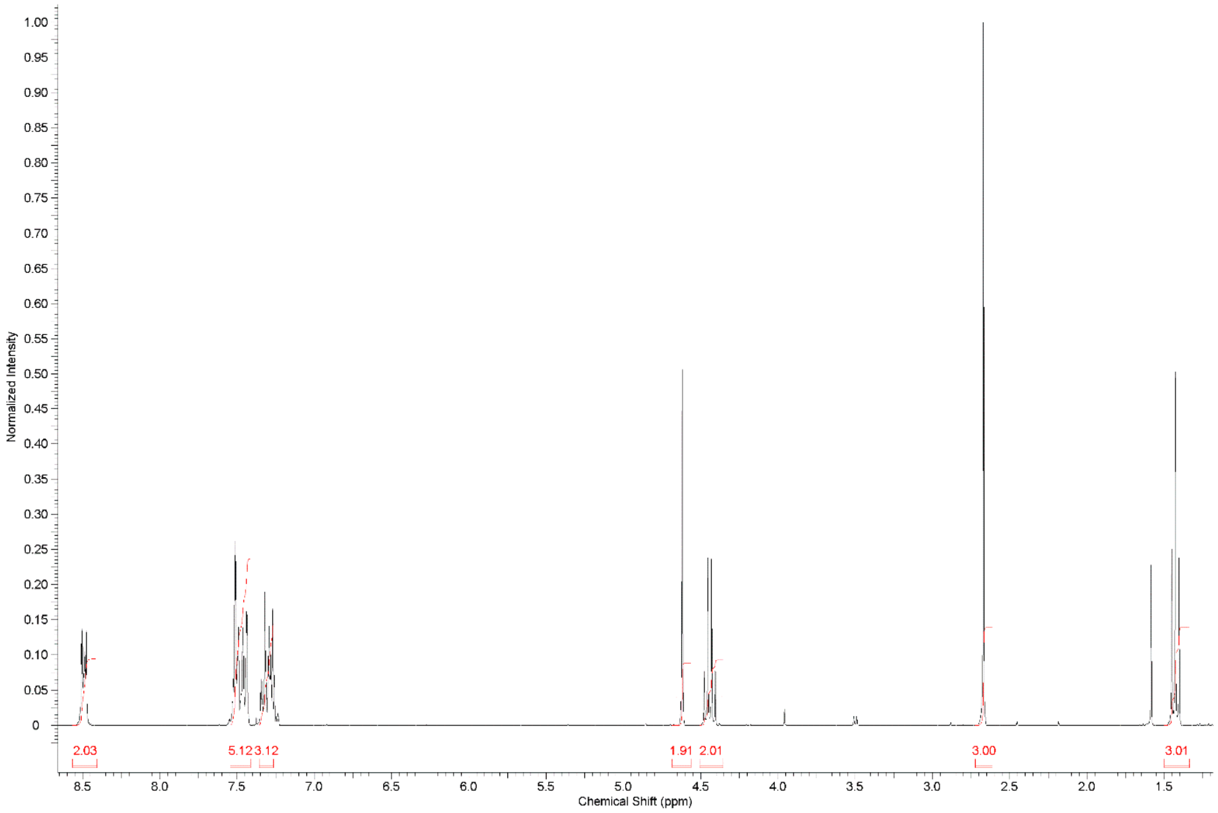

Figure S1. ^{1}HNMR spectrum of compound **2a** (300MHz, $CDCl_3$).

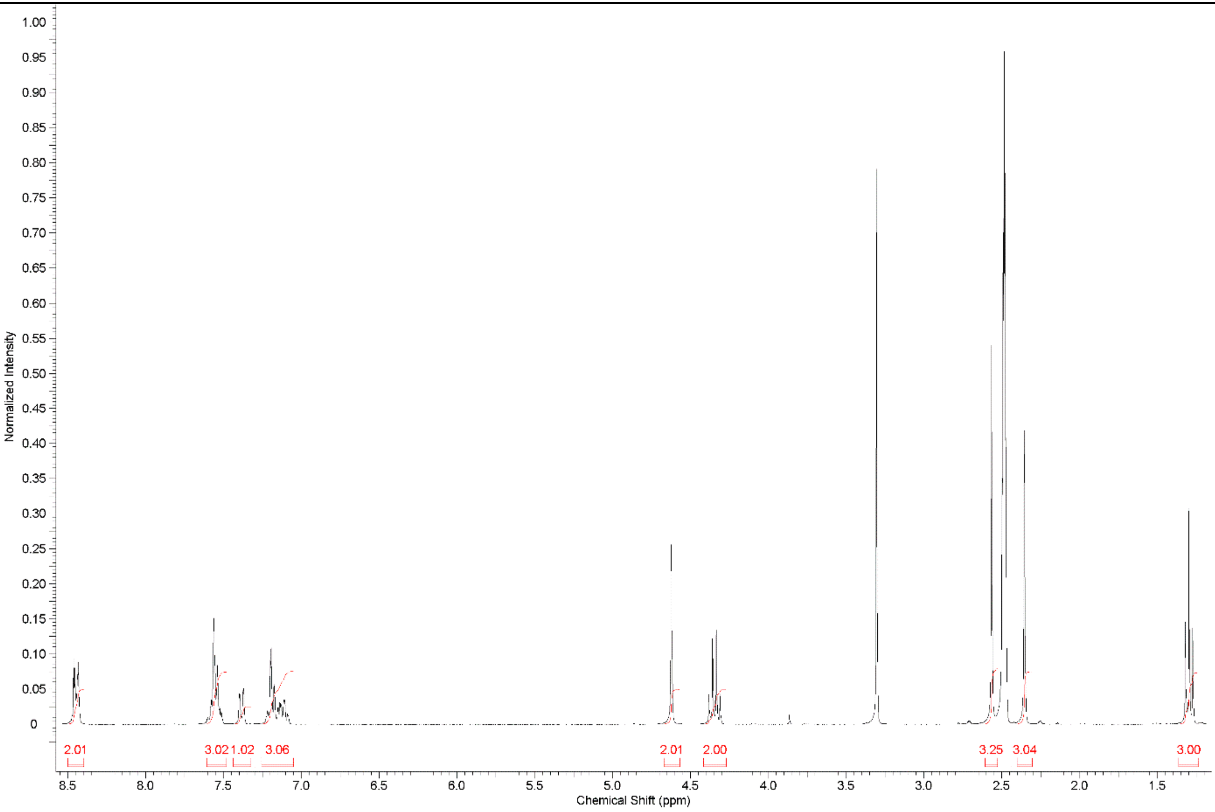

Figure S2. ^{1}HNMR spectrum of compound **2b** (300MHz, DMSO-d_6).

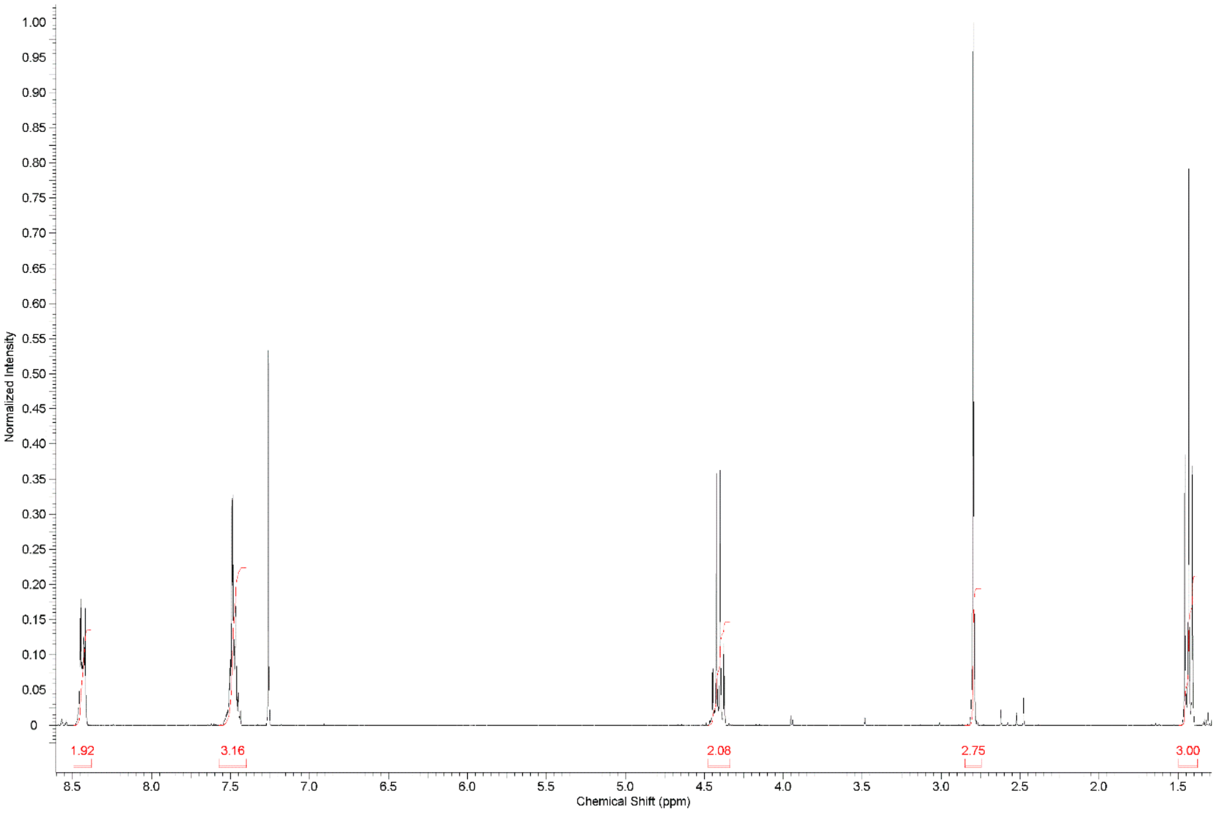

Figure S3. ^{1}HNMR spectrum of compound **2c** (300MHz, $CDCl_3$).

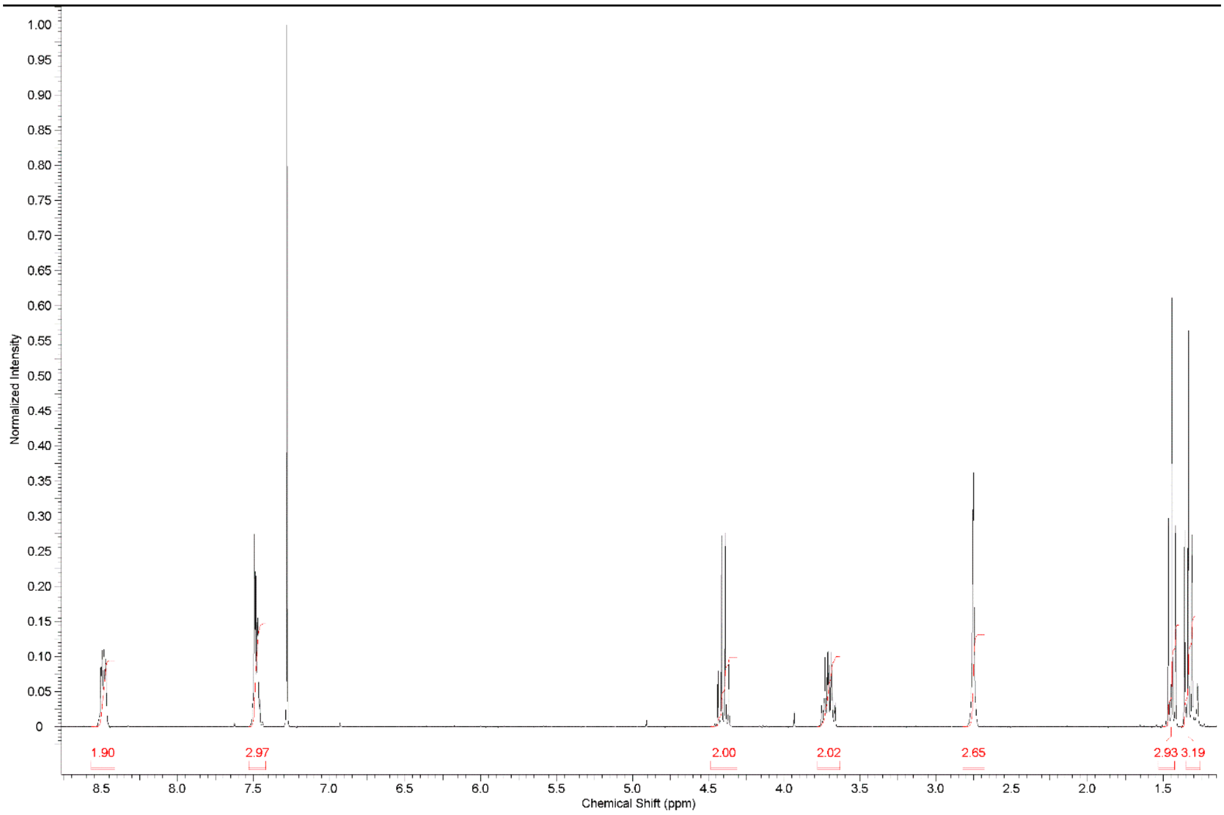

Figure S4. ^{1}HNMR spectrum of compound **2d** (300MHz, $CDCl_3$).

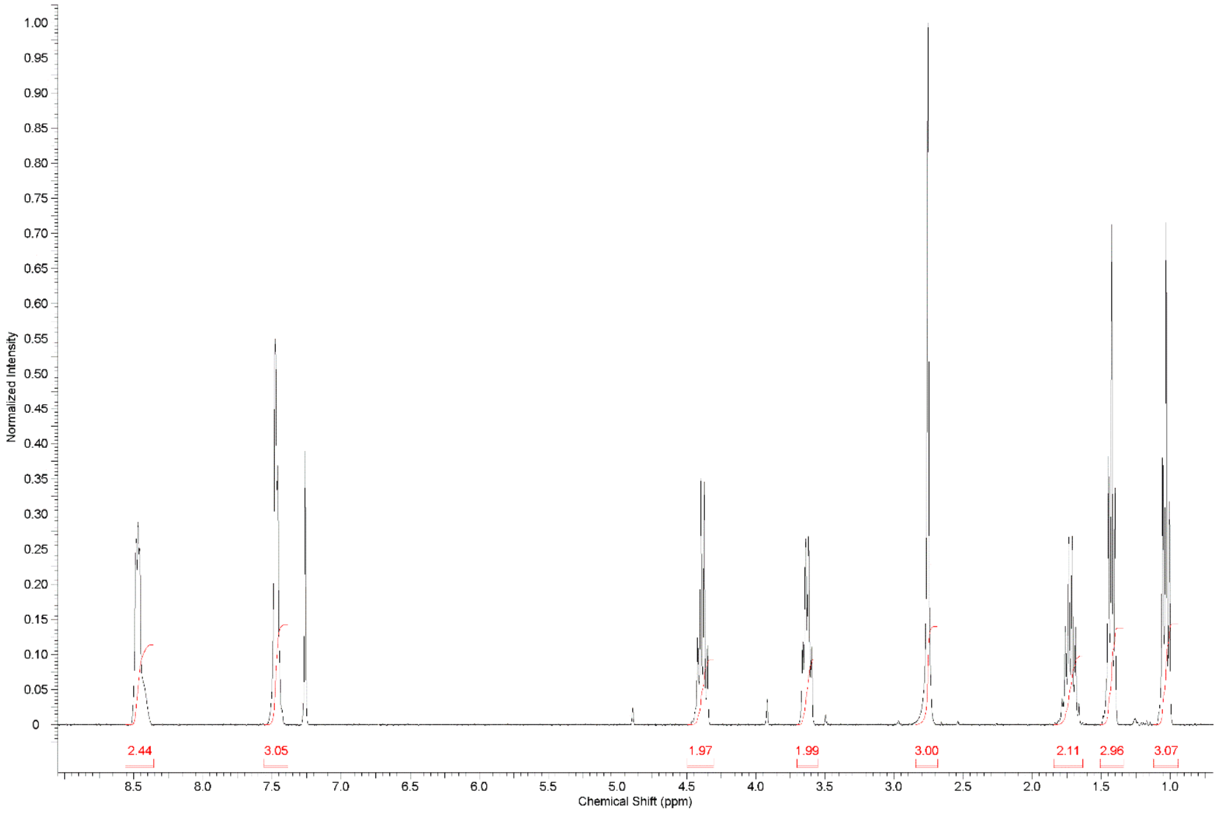

Figure S5. ^{1}HNMR spectrum of compound **2e** (300MHz, $CDCl_3$).

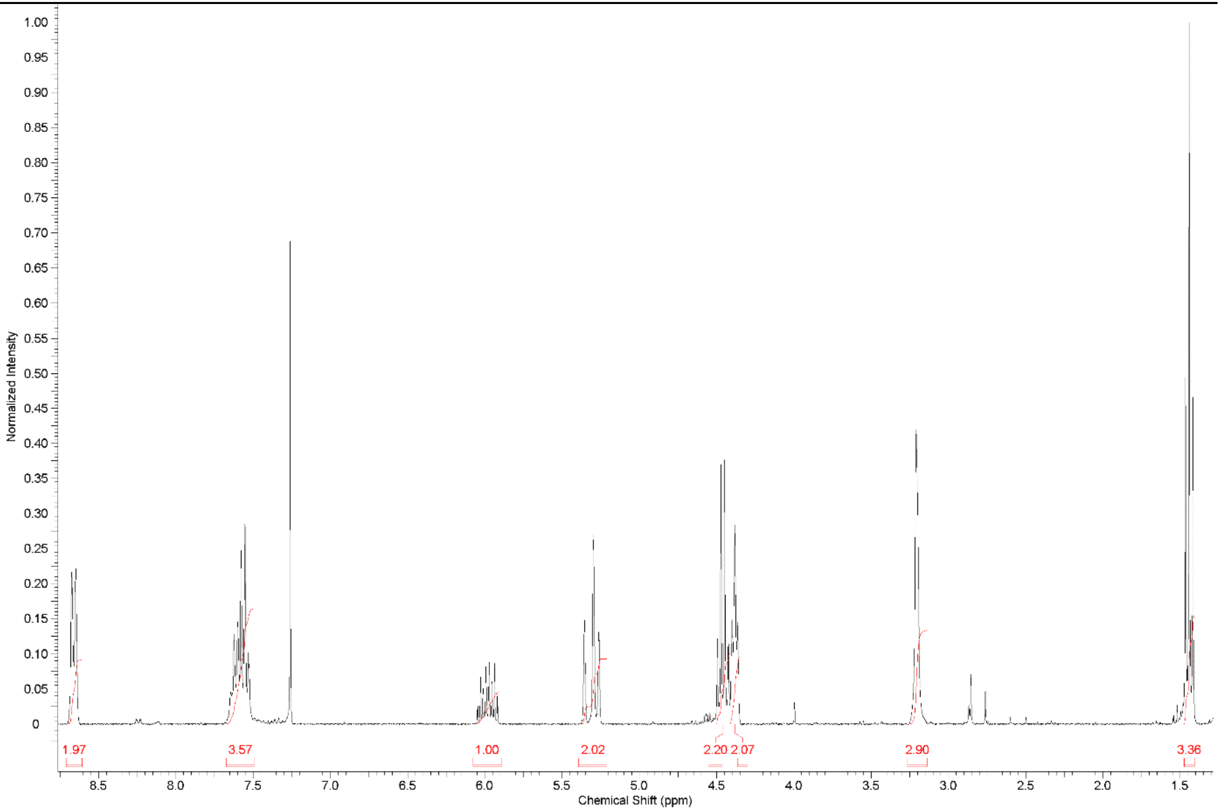

Figure S6. ^{1}HNMR spectrum of compound **2f** (300MHz, $CDCl_3$).

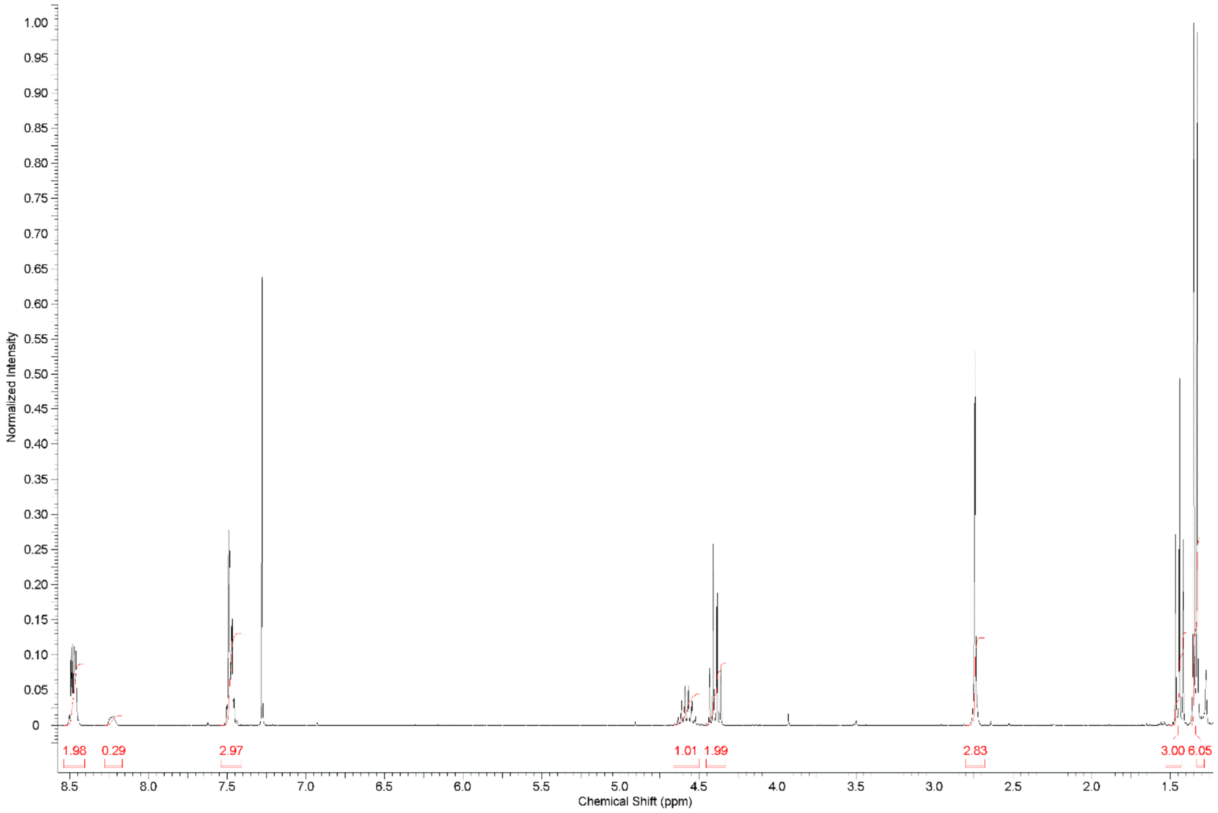

Figure S7. ^{1}HNMR spectrum of compound **2g** (300MHz, $CDCl_3$).

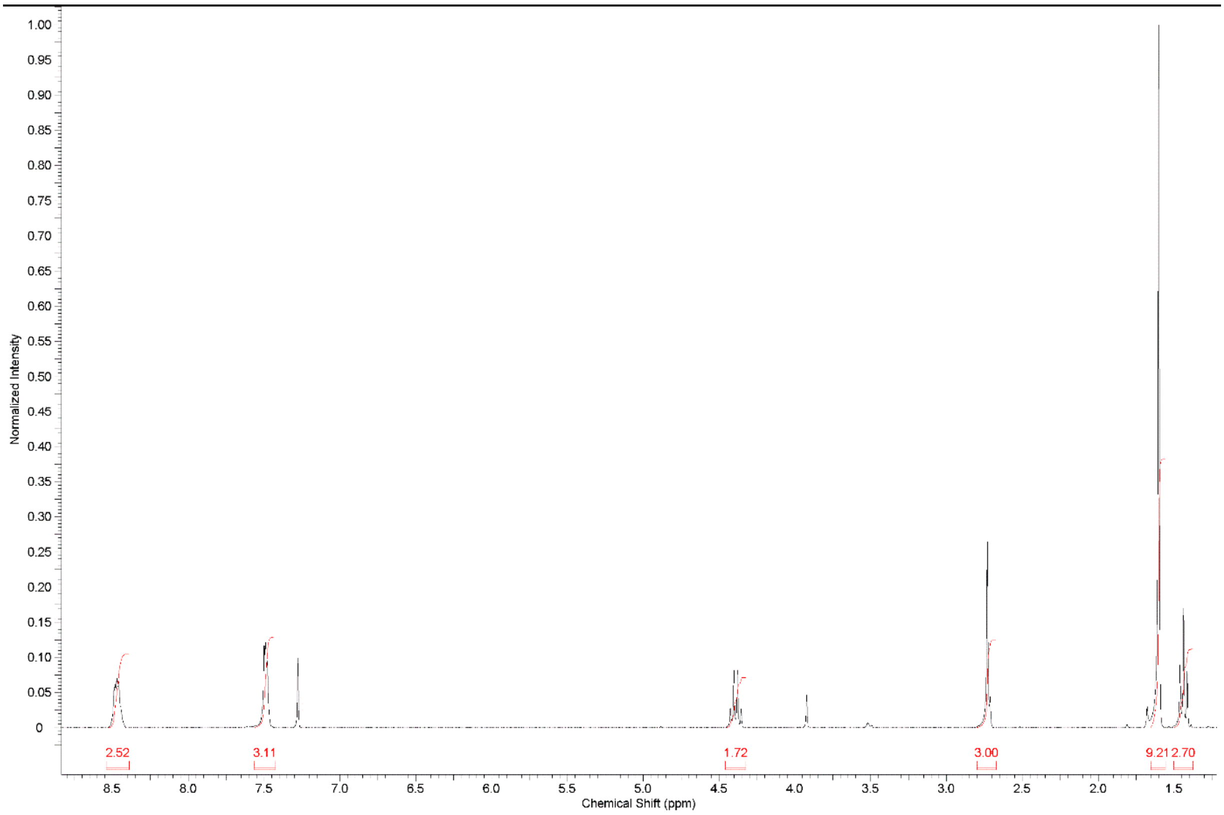

Figure S8. ^{1}HNMR spectrum of compound **2h** (300MHz, $CDCl_3$).

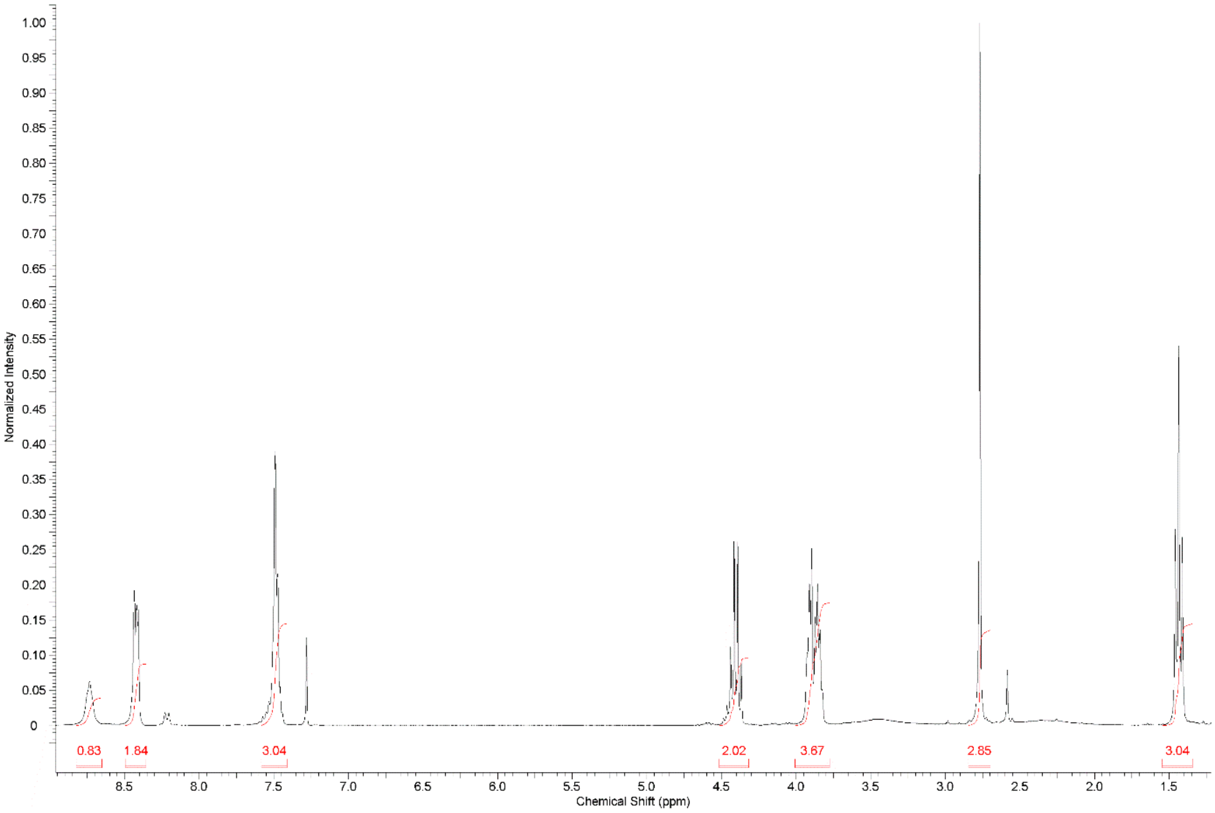

Figure S9. ^{1}HNMR spectrum of compound **2i** (300MHz, $CDCl_3$).

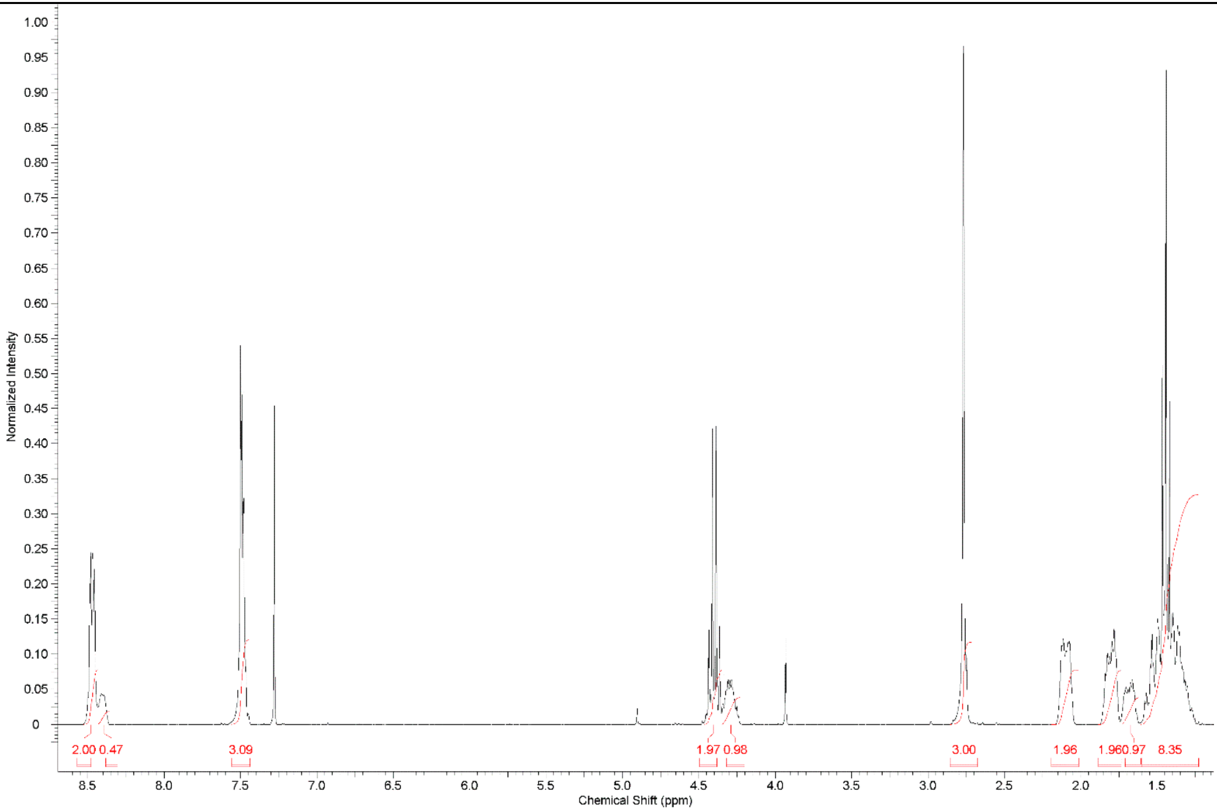

Figure S10. ^{1}HNMR spectrum of compound **2j** (300MHz, $CDCl_3$).

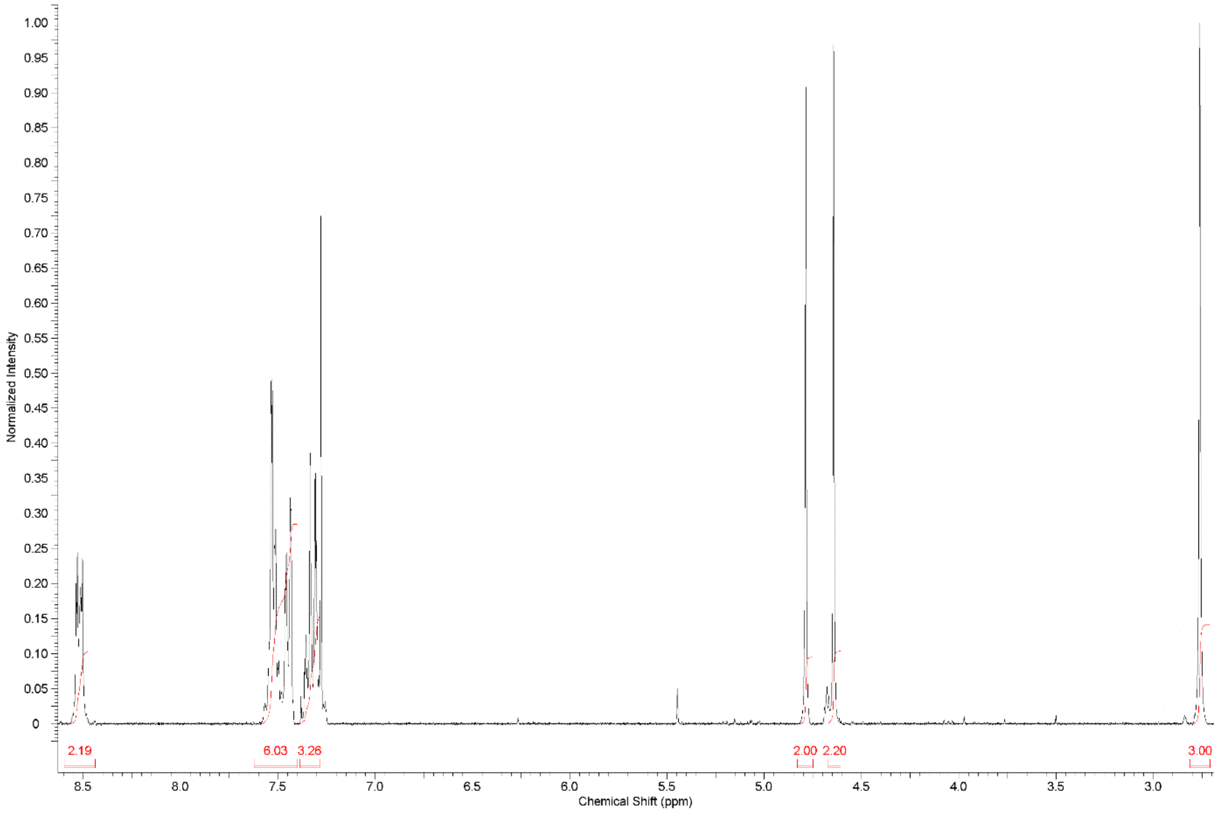

Figure S11. ^{1}HNMR spectrum of compound **3a** (300MHz, $CDCl_3$).

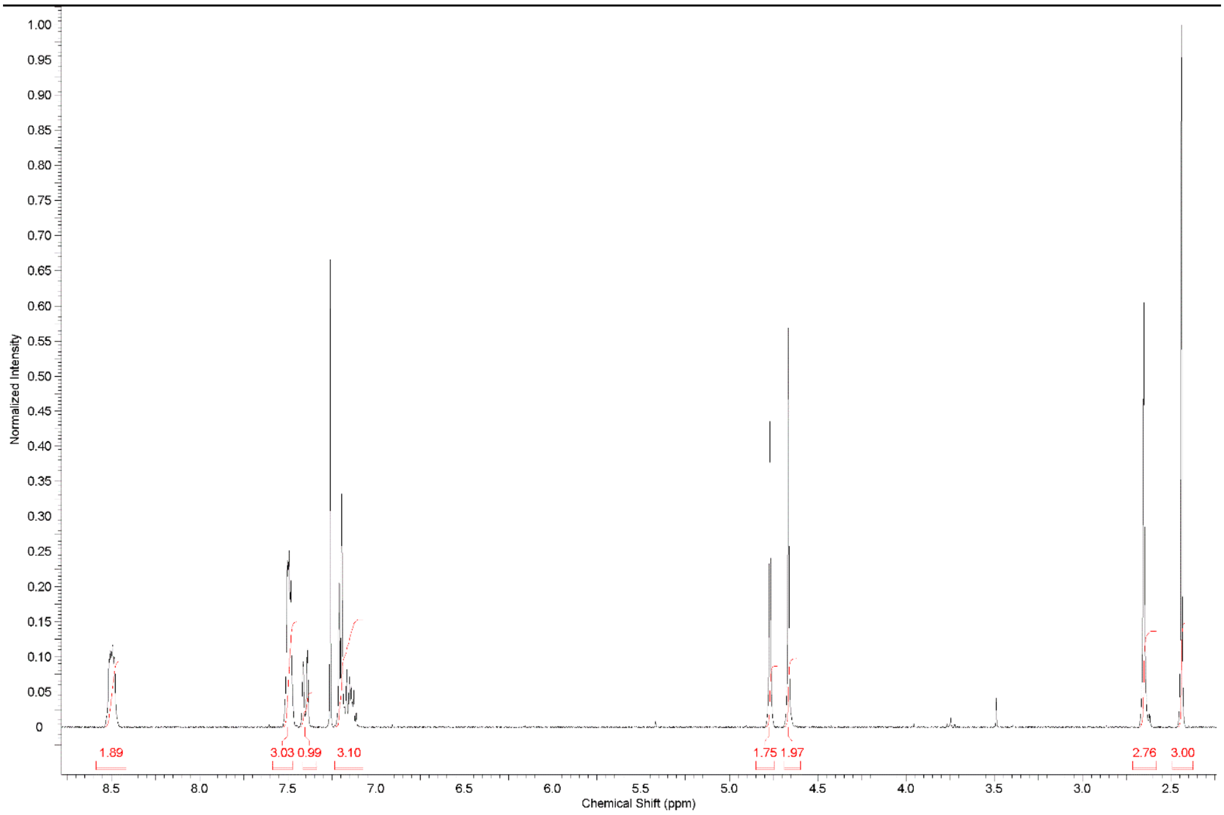

Figure S12. ^{1}HNMR spectrum of compound **3b** (300MHz, $CDCl_3$).

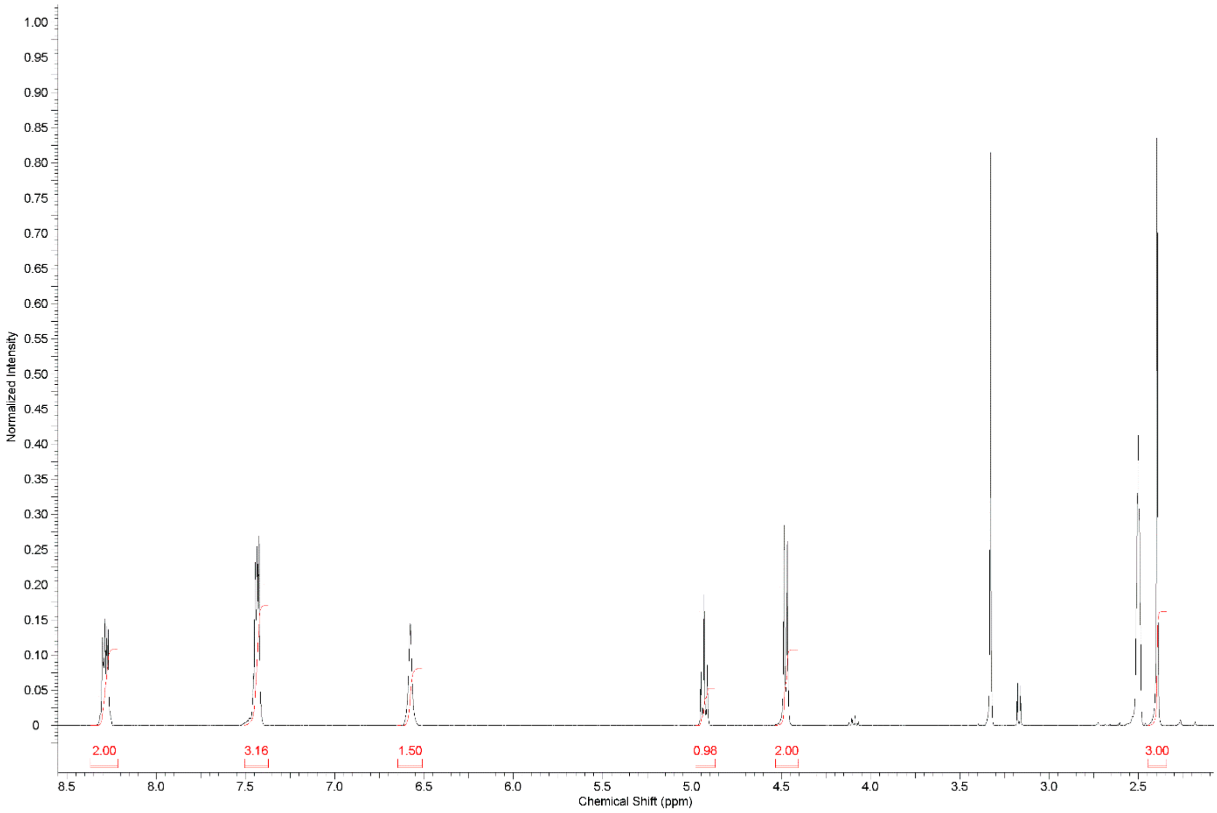

Figure S13. ^{1}HNMR spectrum of compound **3c** (300MHz, DMSO-d_6).

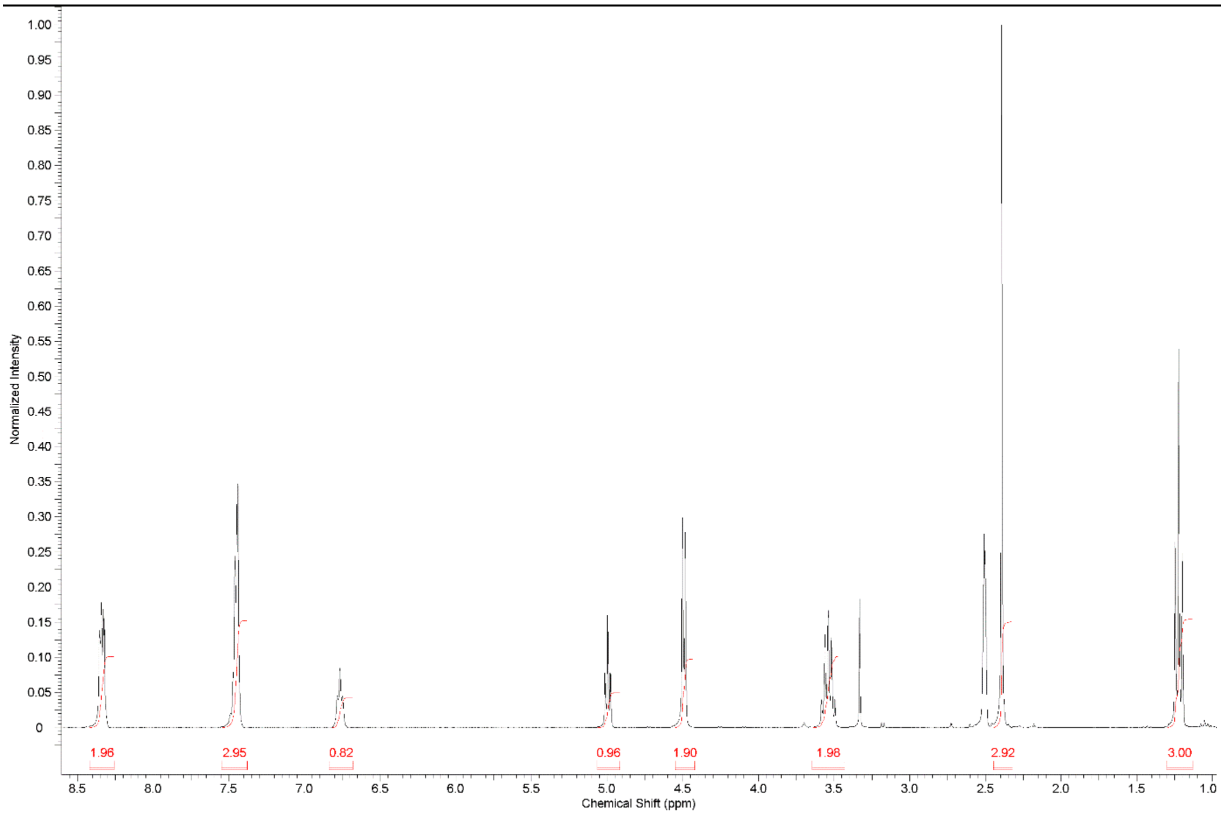

Figure S14. ^{1}HNMR spectrum of compound **3d** (300MHz, DMSO-d_6).

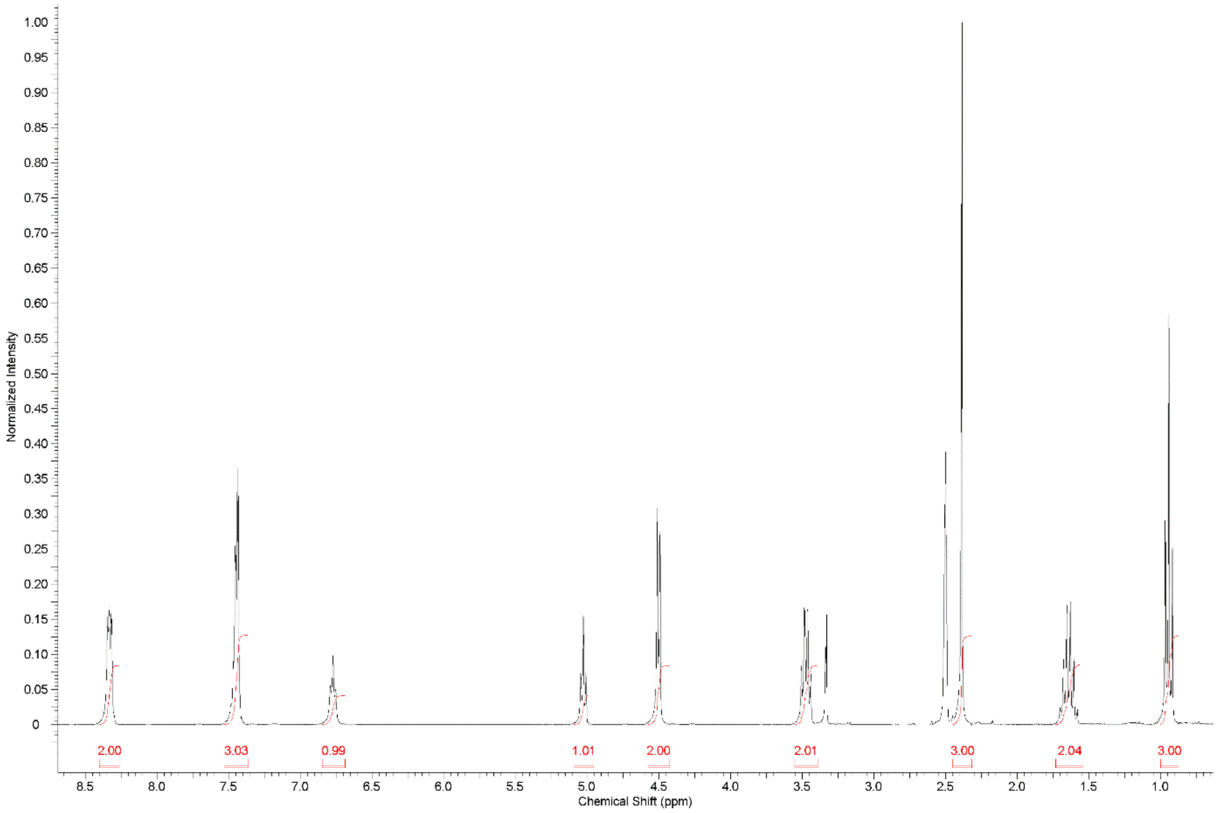

Figure S15. ^{1}HNMR spectrum of compound **3e** (300MHz, DMSO-d_6).

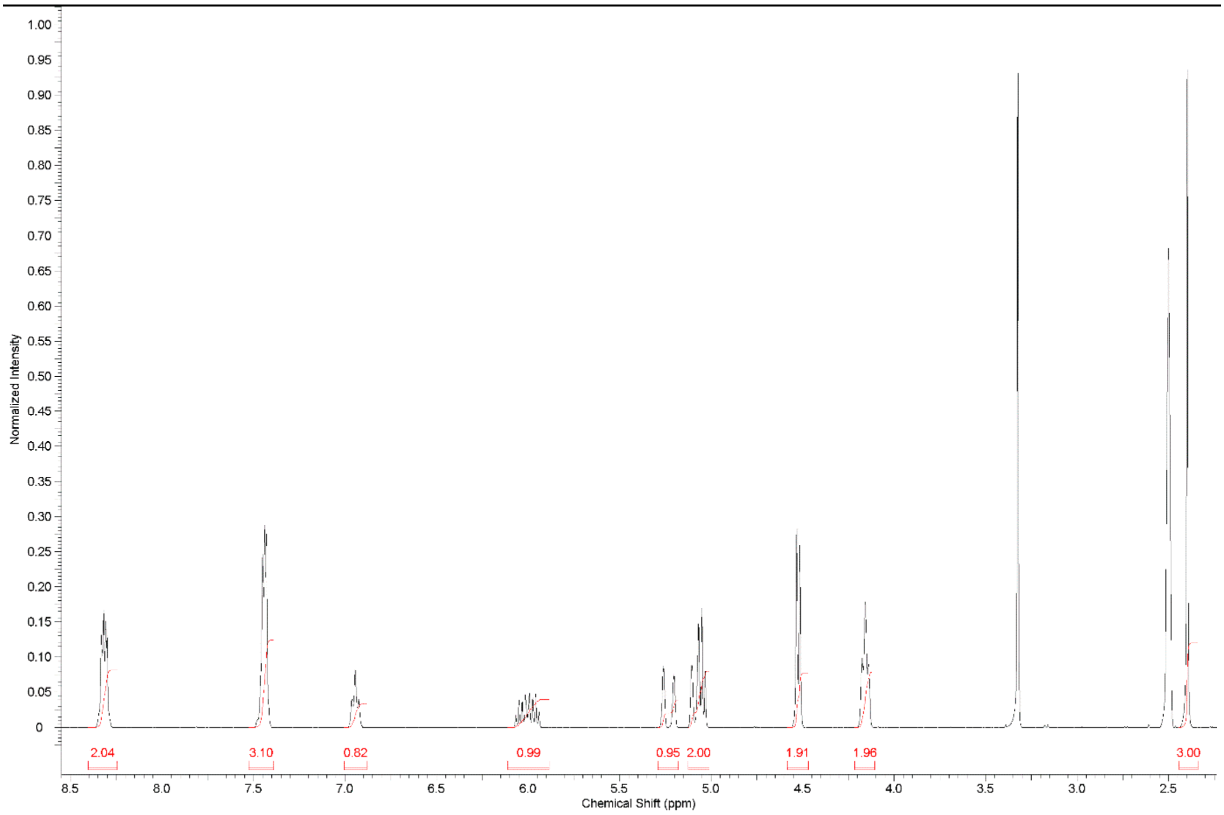

Figure S16. ^{1}HNMR spectrum of compound **3f** (300MHz, DMSO-d_6).

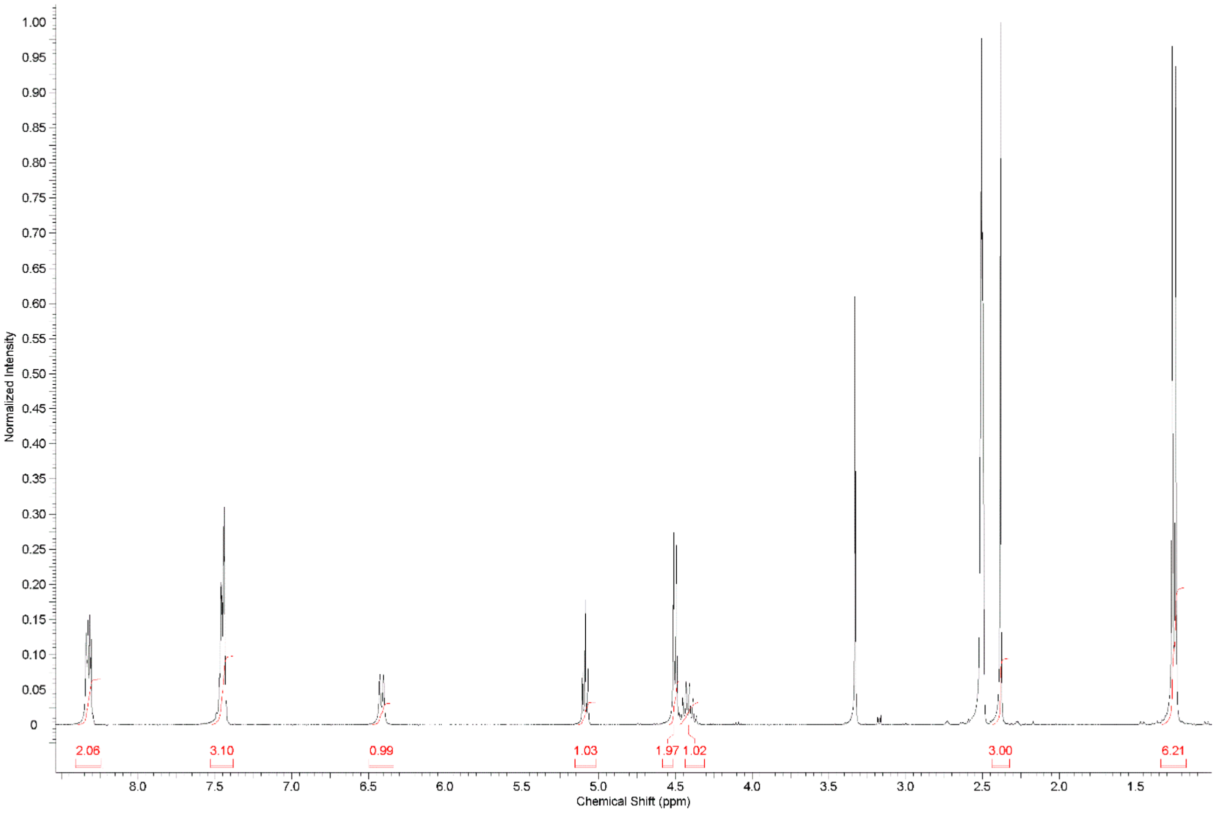

Figure S17. ^{1}HNMR spectrum of compound **3g** (300MHz, DMSO-d_6).

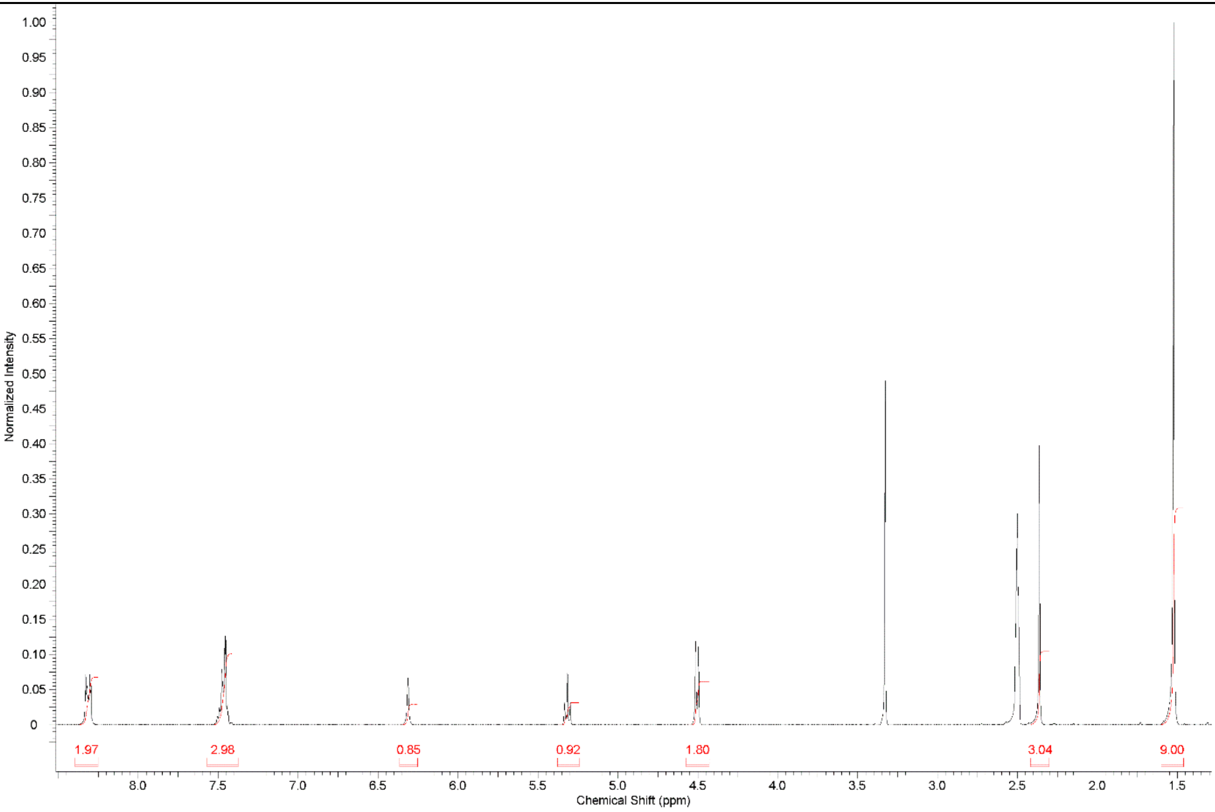

Figure S18. ^{1}HNMR spectrum of compound **3h** (300MHz, DMSO-d$_6$).

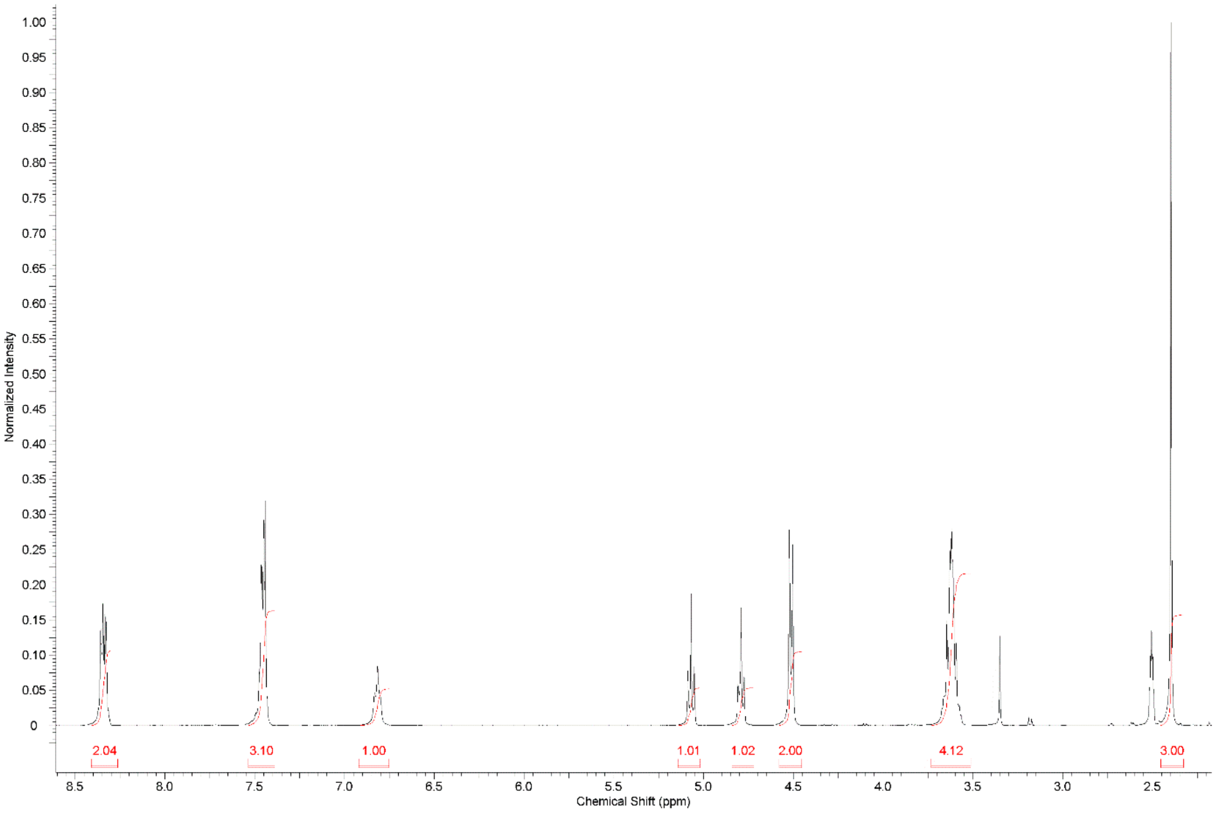

Figure S19. ^{1}HNMR spectrum of compound **3i** (300MHz, DMSO-d$_6$).

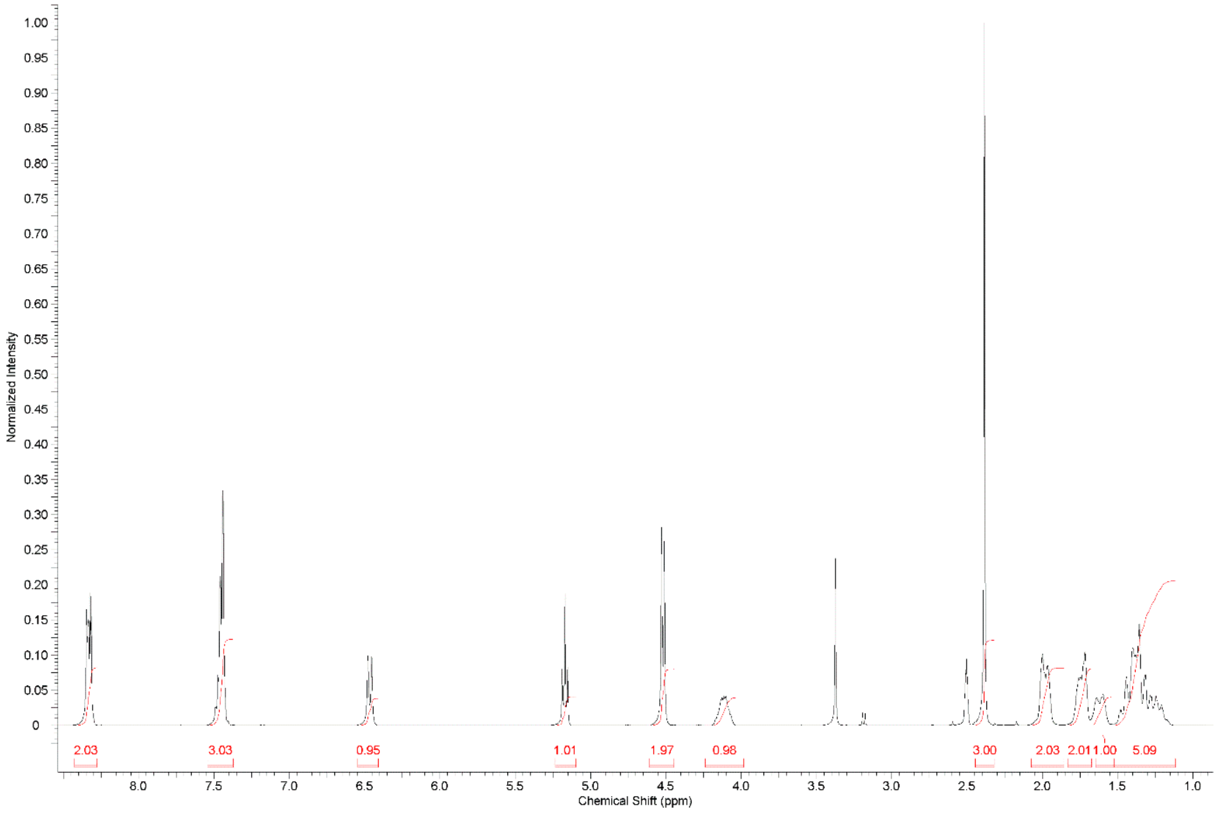

Figure S20. ^{1}HNMR spectrum of compound **3j** (300MHz, DMSO-d_6).

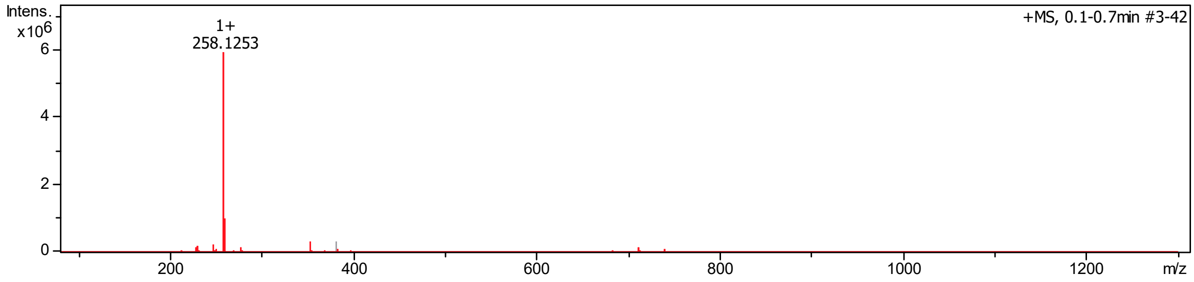

Figure S21. ESI-MS spectrum of compound **2a**.

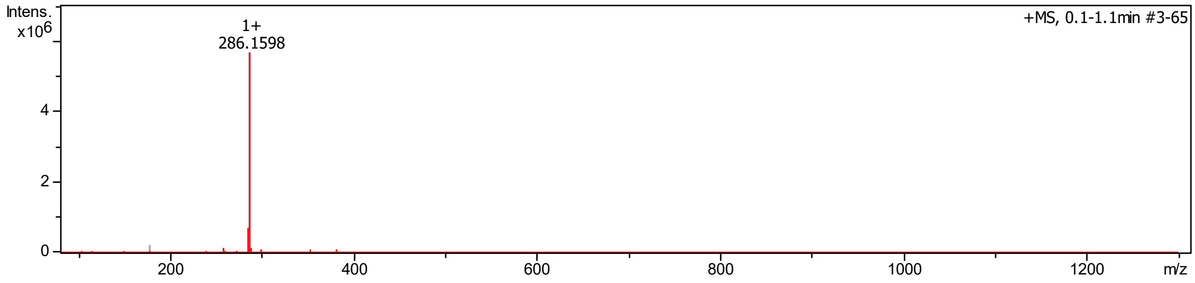

Figure S22. ESI-MS spectrum of compound **2b**.

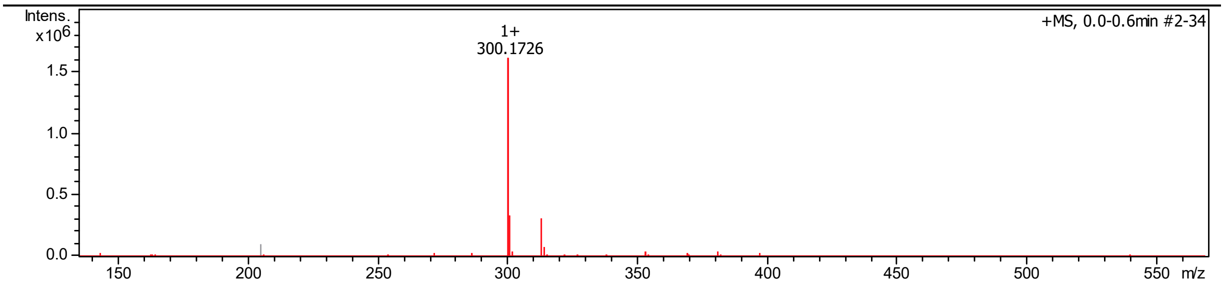

Figure S23. ESI-MS spectrum of compound **2c**.

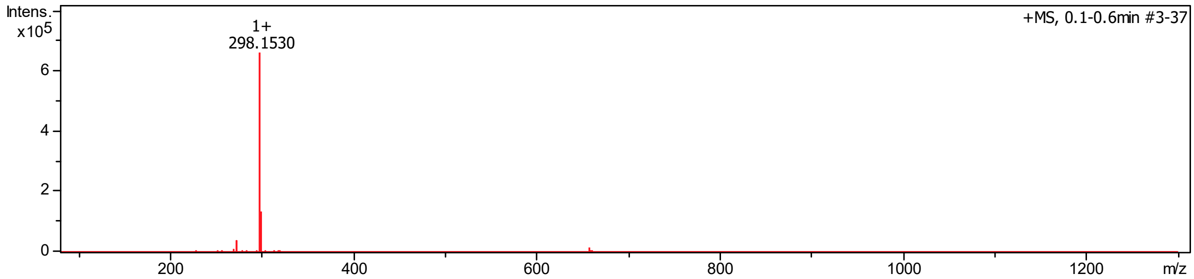

Figure S24. ESI-MS spectrum of compound **2d**.

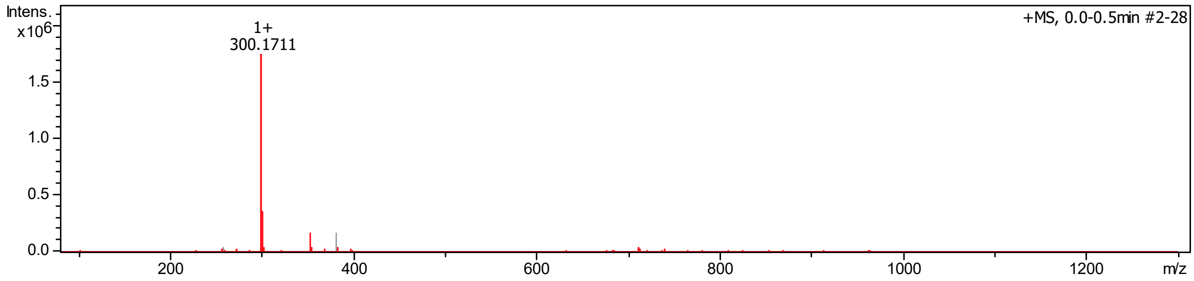

Figure S25. ESI-MS spectrum of compound **2e**.

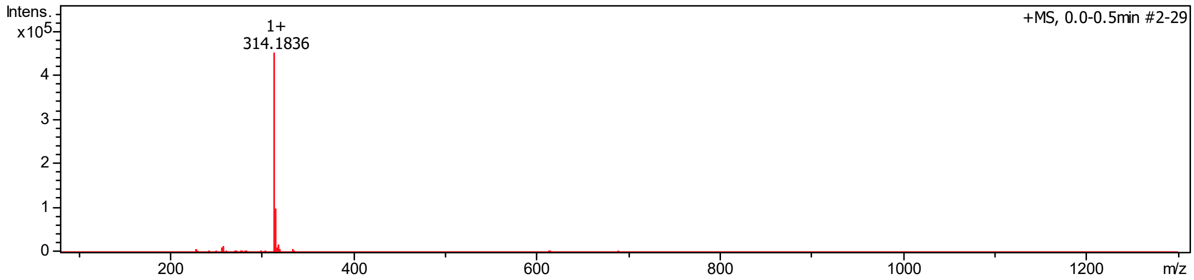

Figure S26. ESI-MS spectrum of compound **2f**.

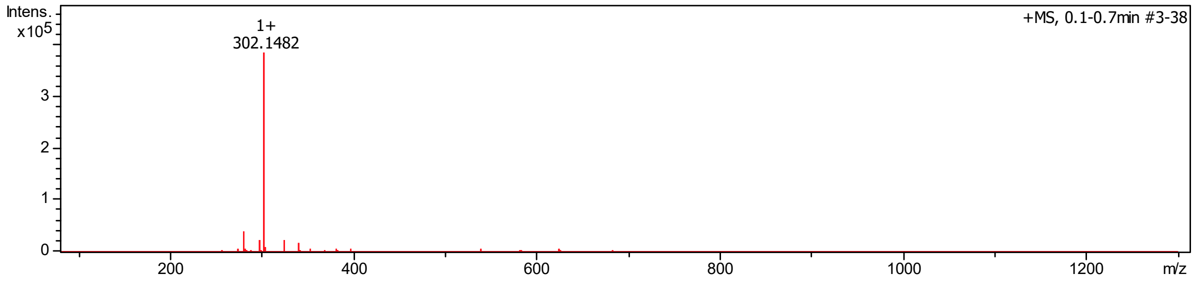

Figure S27. ESI-MS spectrum of compound **2g**.

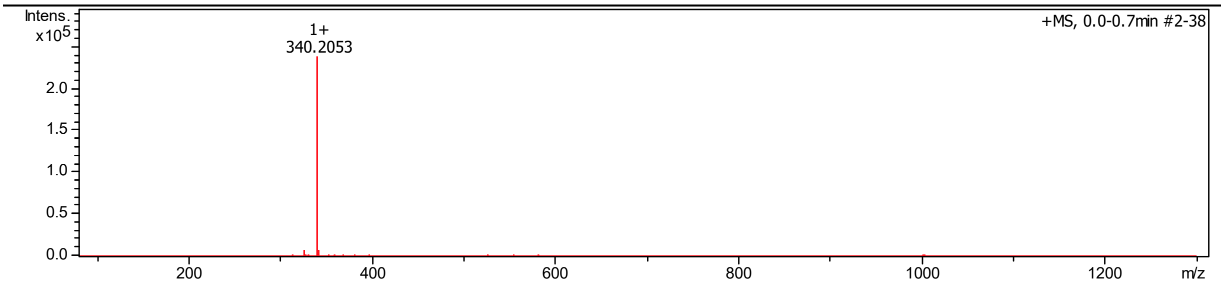

Figure S28. ESI-MS spectrum of compound **2h**.

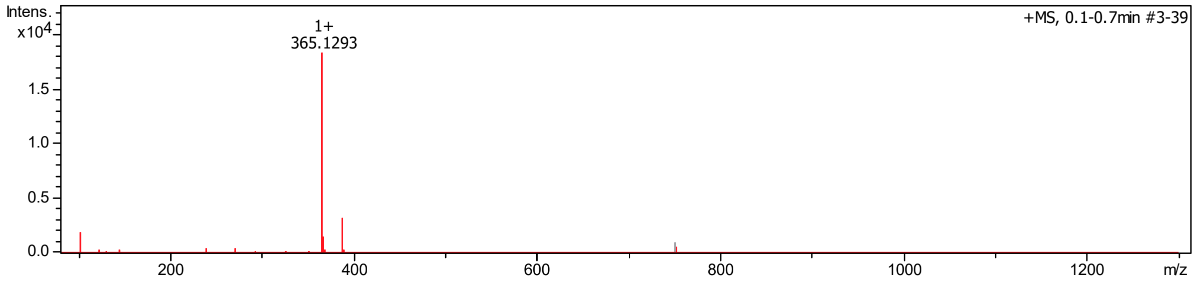

Figure S29. ESI-MS spectrum of compound **2i**.

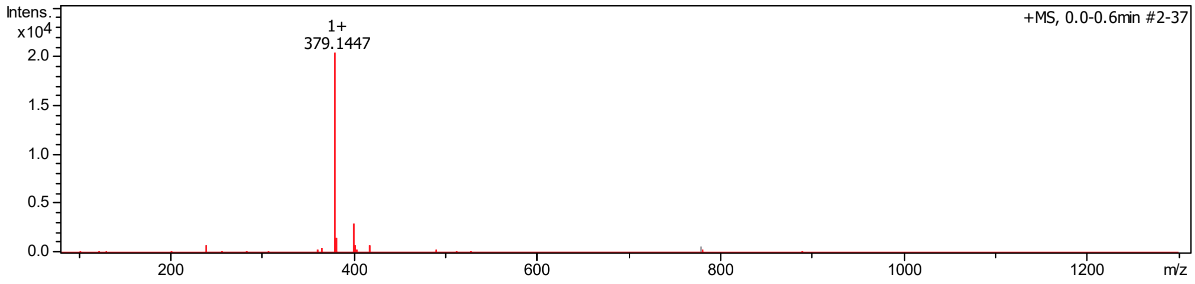

Figure S30. ESI-MS spectrum of compound **2j**.

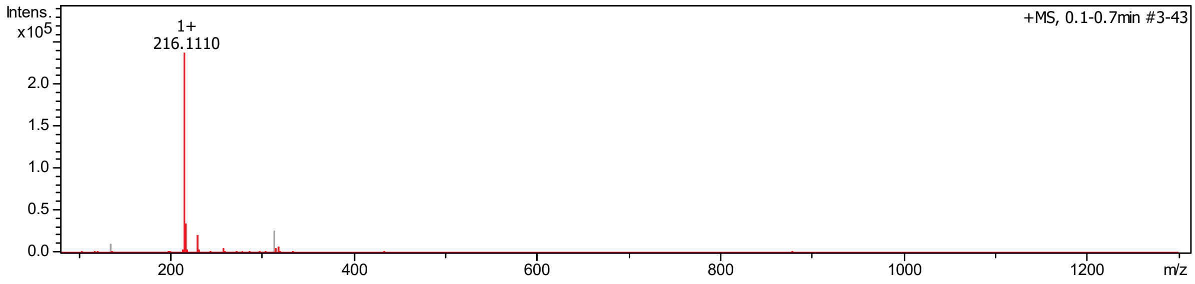

Figure S31. ESI-MS spectrum of compound **3a**.

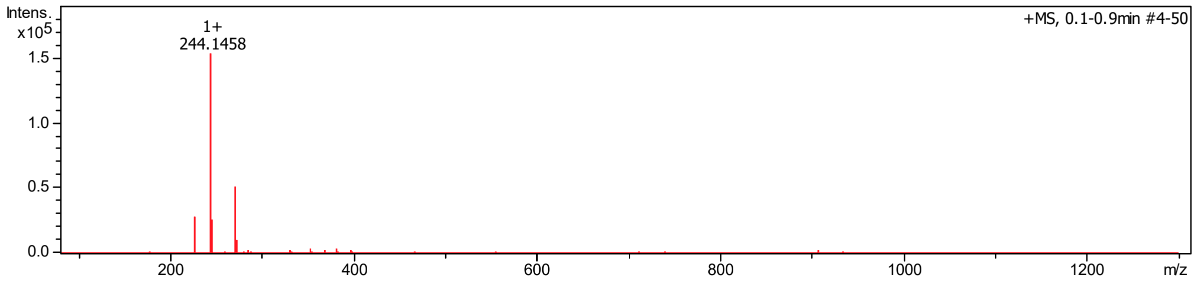

Figure S32. ESI-MS spectrum of compound **3b**.

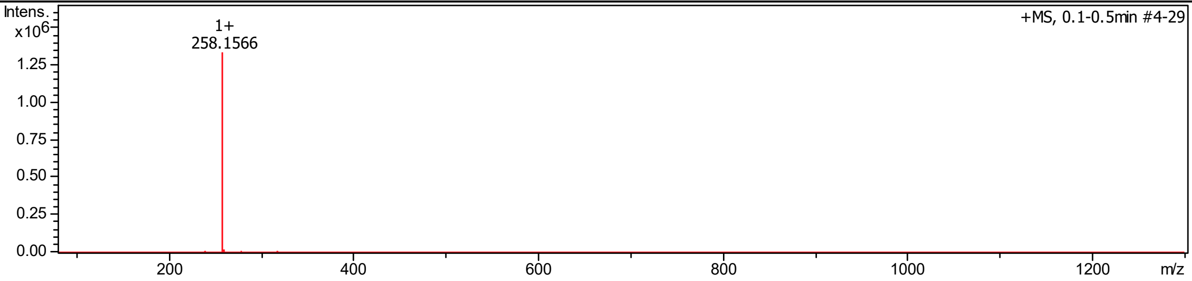

Figure S33. ESI-MS spectrum of compound **3c**.

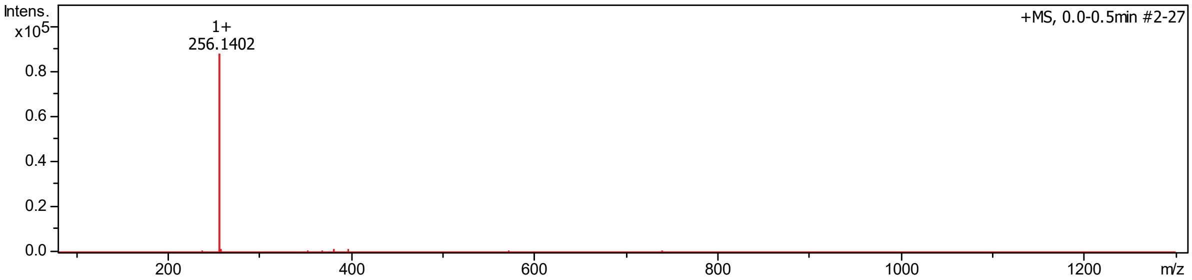

Figure S34. ESI-MS spectrum of compound **3d**.

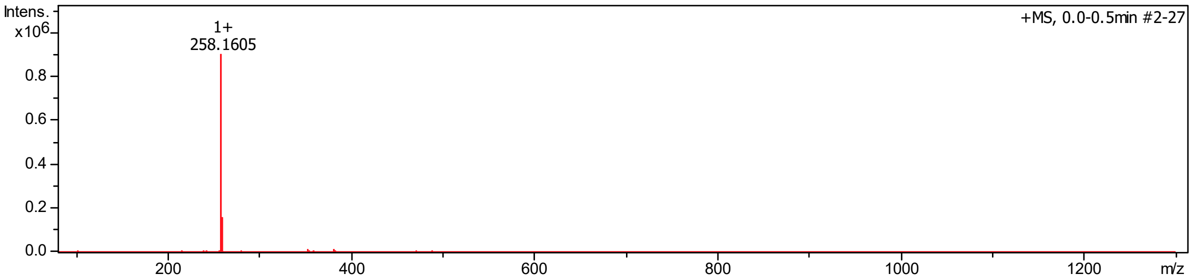

Figure S35. ESI-MS spectrum of compound **3e**.

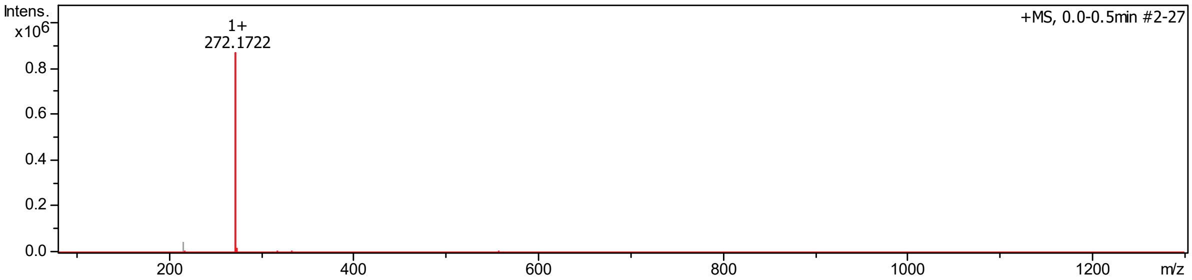

Figure S36. ESI-MS spectrum of compound **3f**.

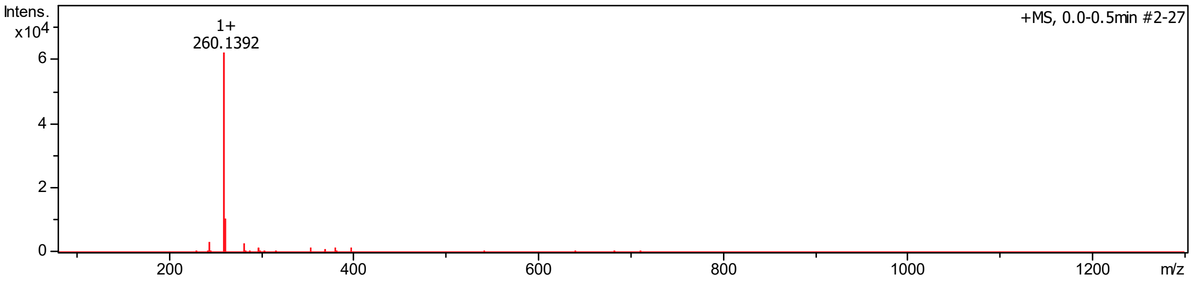

Figure S37. ESI-MS spectrum of compound **3g**.

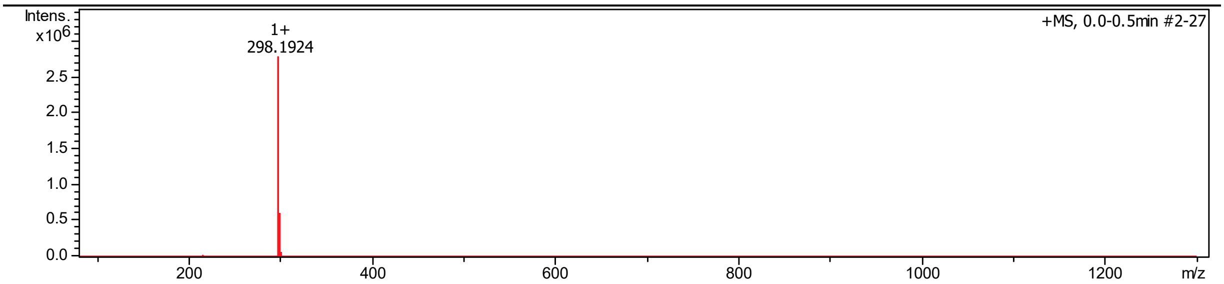

Figure S38. ESI-MS spectrum of compound **3h**.

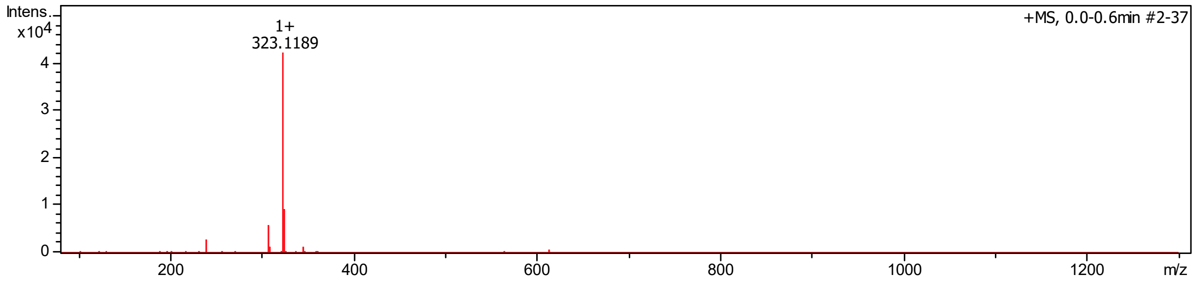

Figure S39. ESI-MS spectrum of compound **3i**.

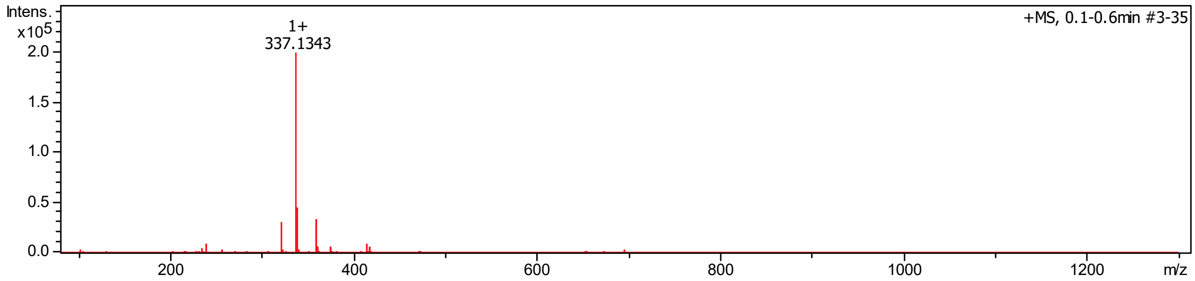

Figure S40. ESI-MS spectrum of compound **3j**.

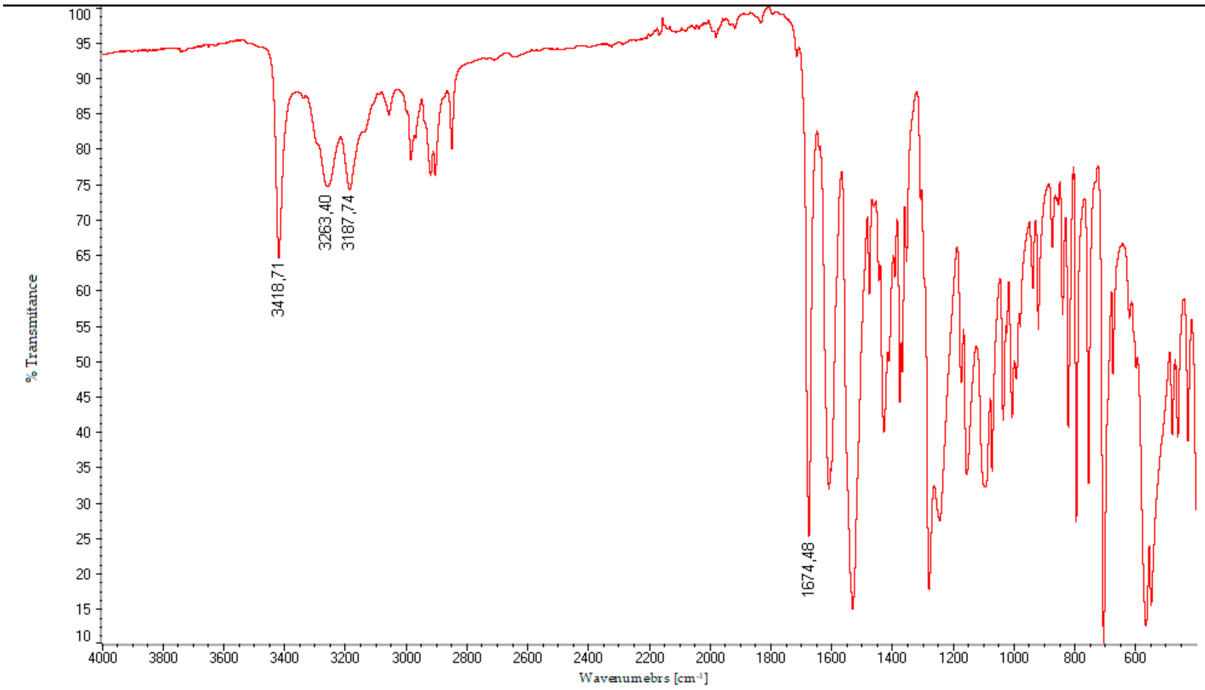

Figure S41. IR spectrum of compound **2a**.

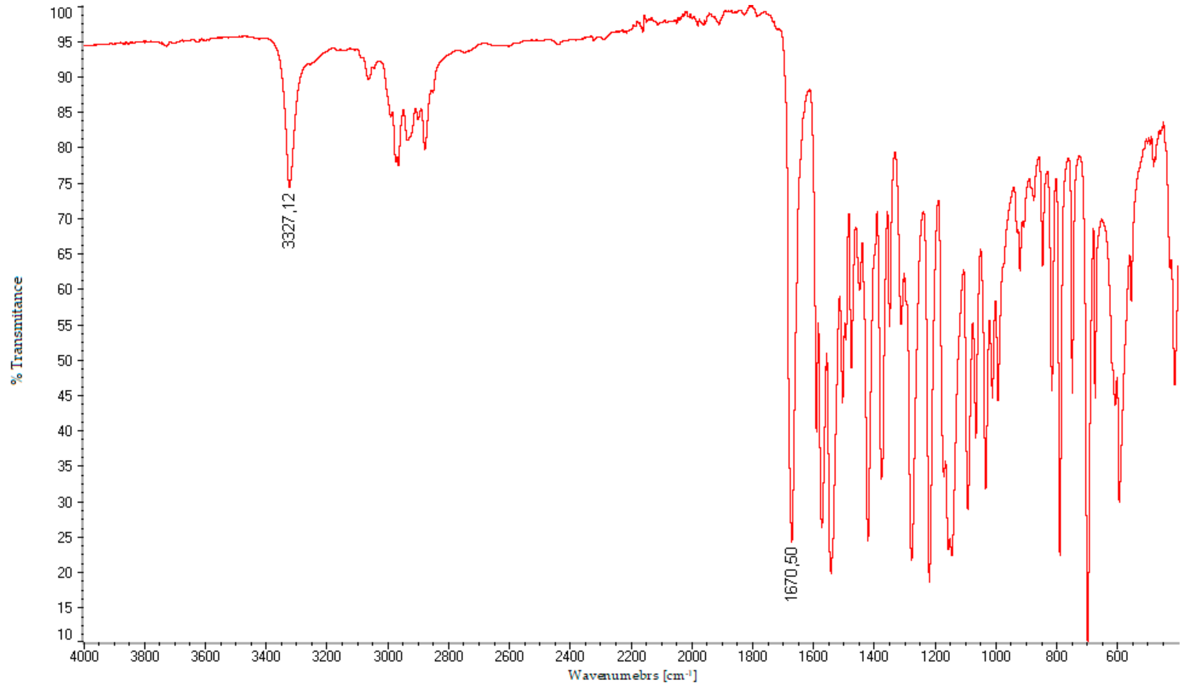

Figure S42. IR spectrum of compound **2b**.

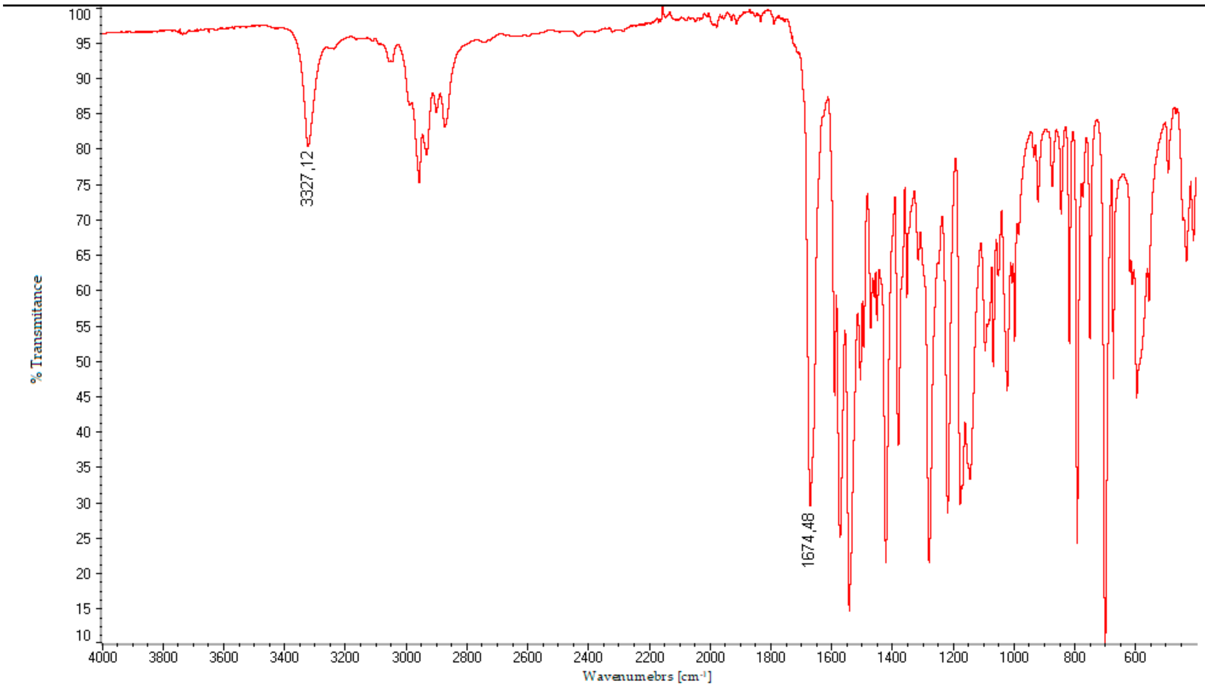

Figure S43. IR spectrum of compound **2c**.

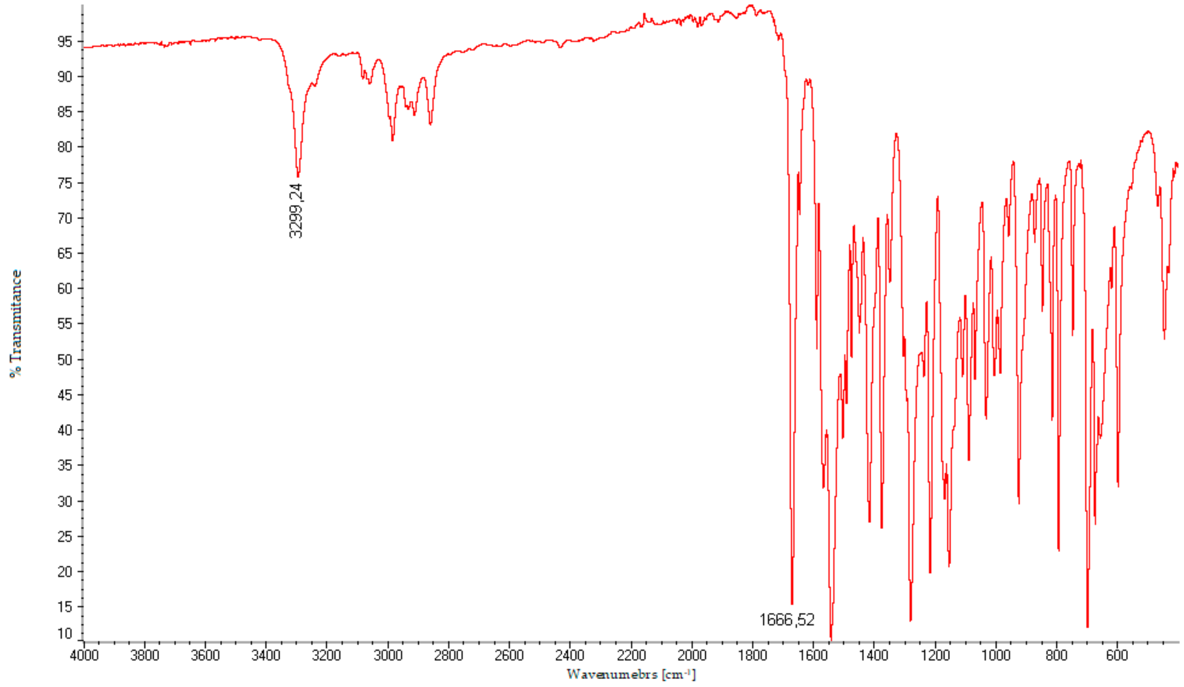

Figure S44. IR spectrum of compound **2d**.

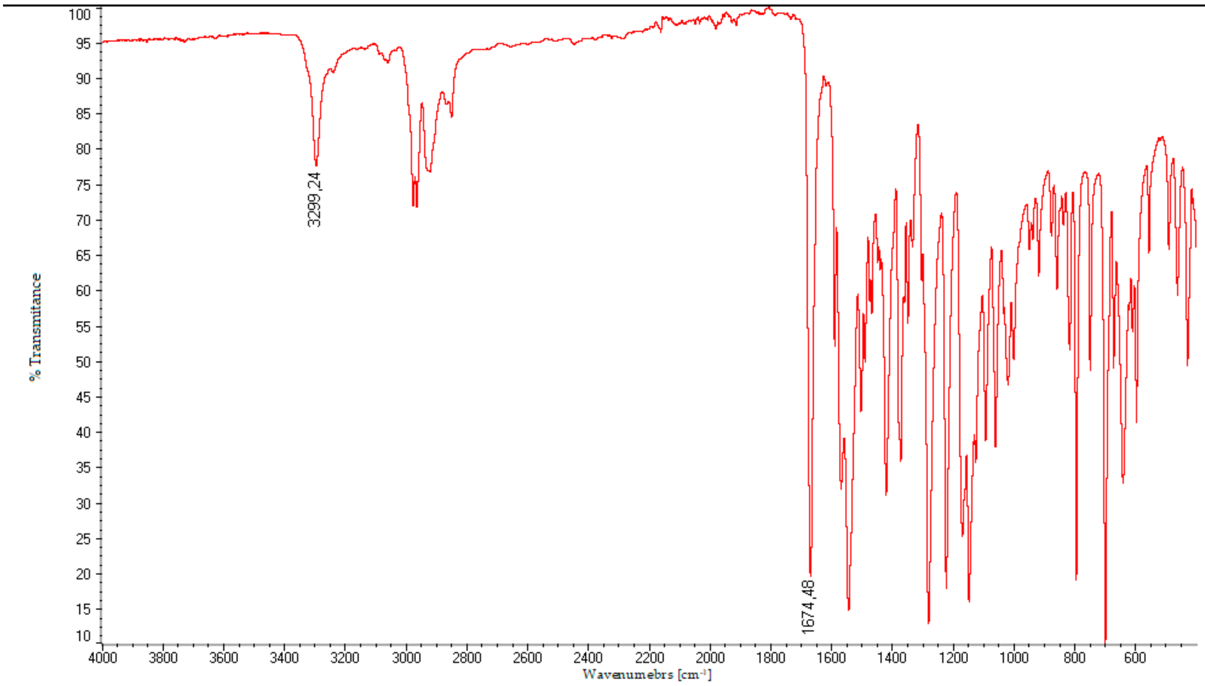

Figure S45. IR spectrum of compound **2e**.

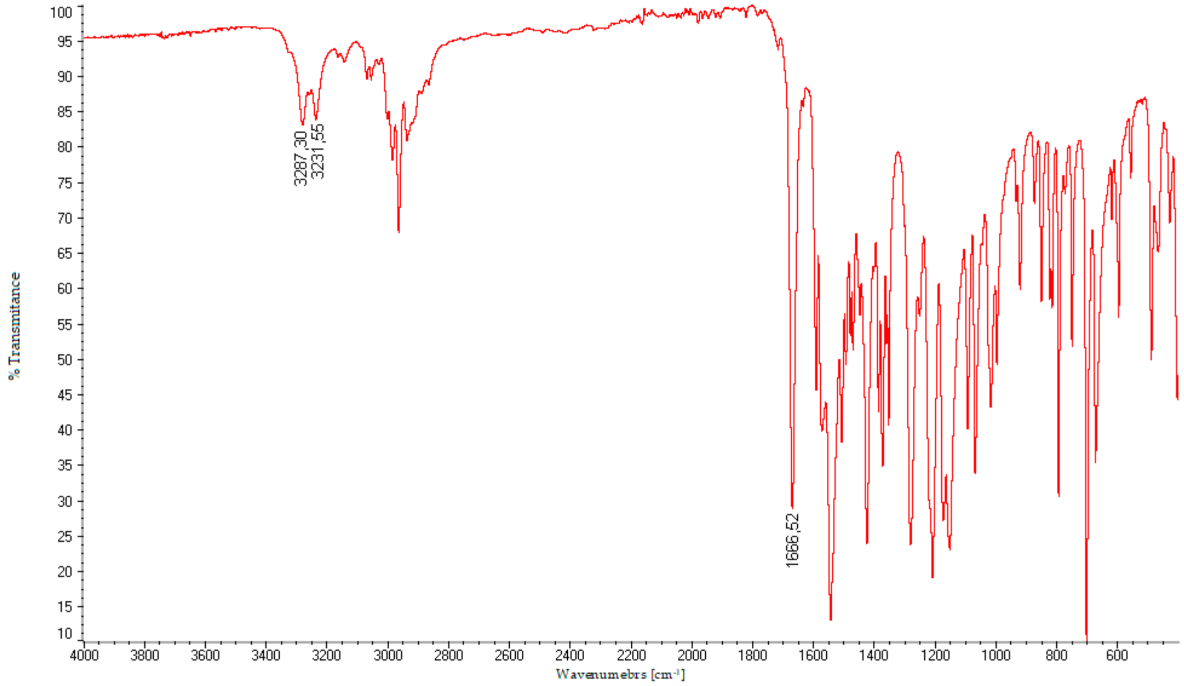

Figure S46. IR spectrum of compound **2f**.

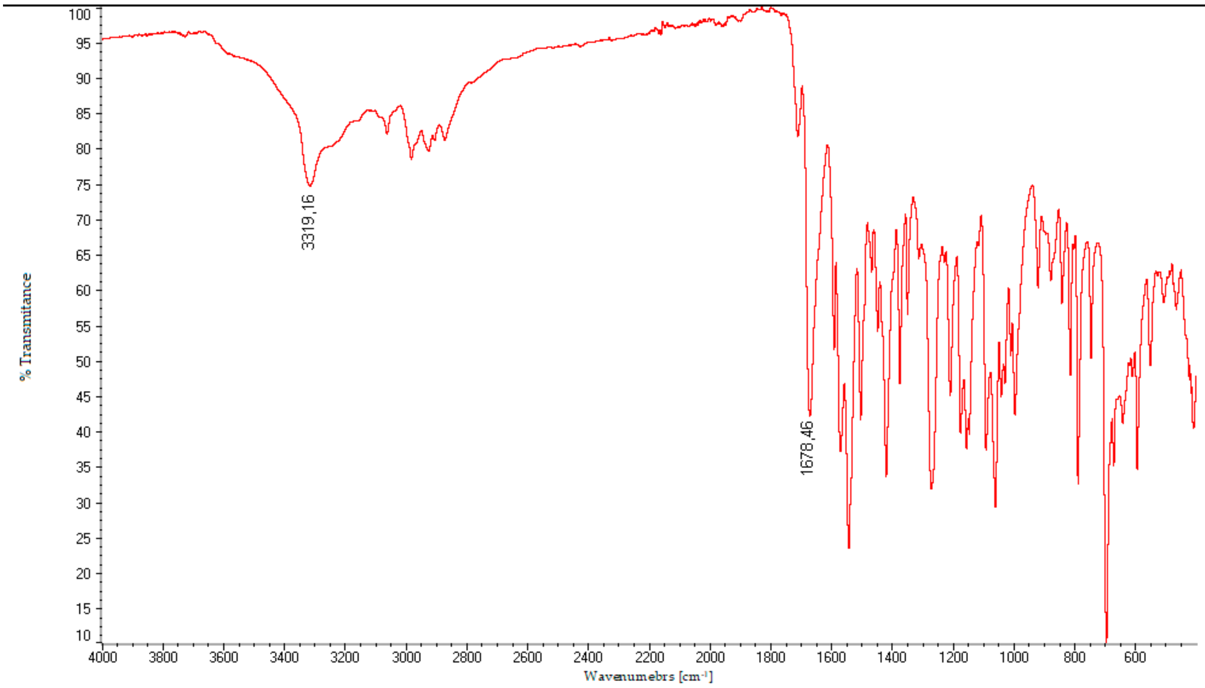

Figure S47. IR spectrum of compound **2g**.

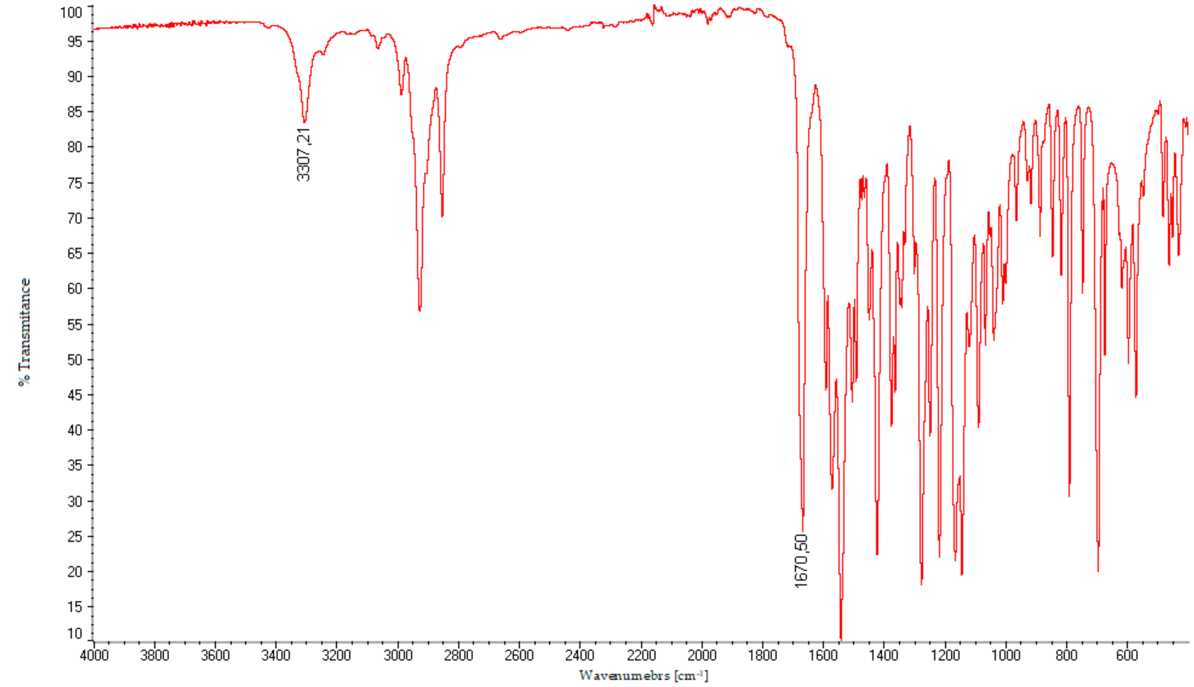

Figure S48. IR spectrum of compound **2h**.

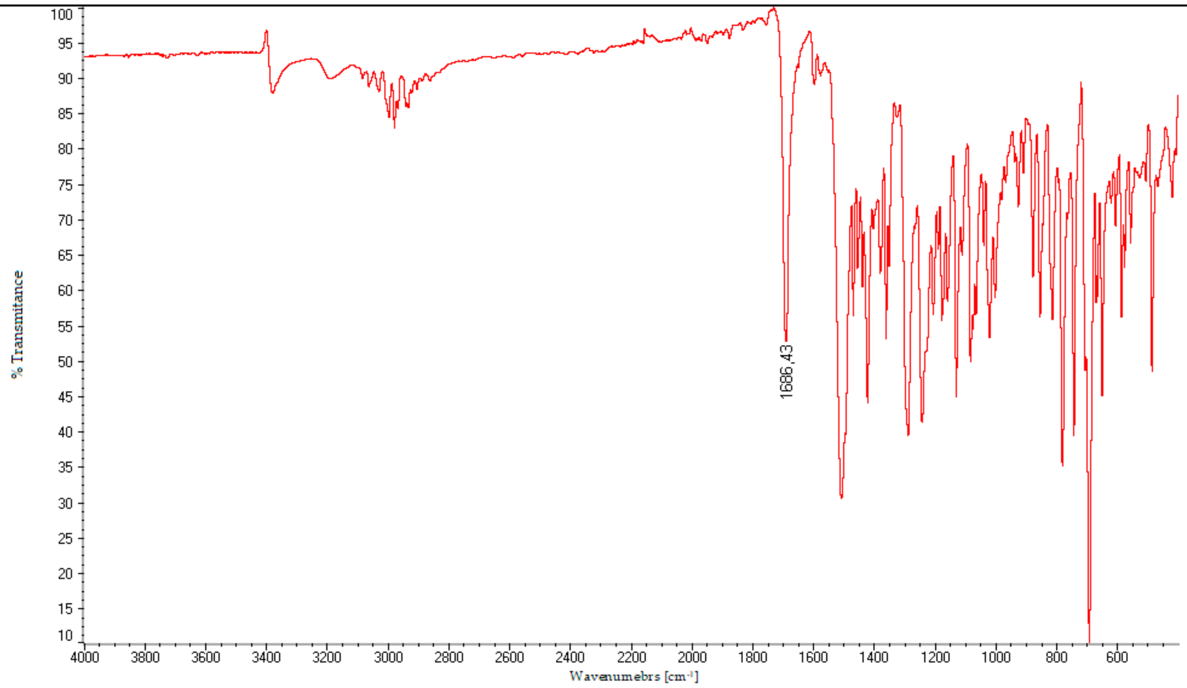

Figure S49. IR spectrum of compound **2i**.

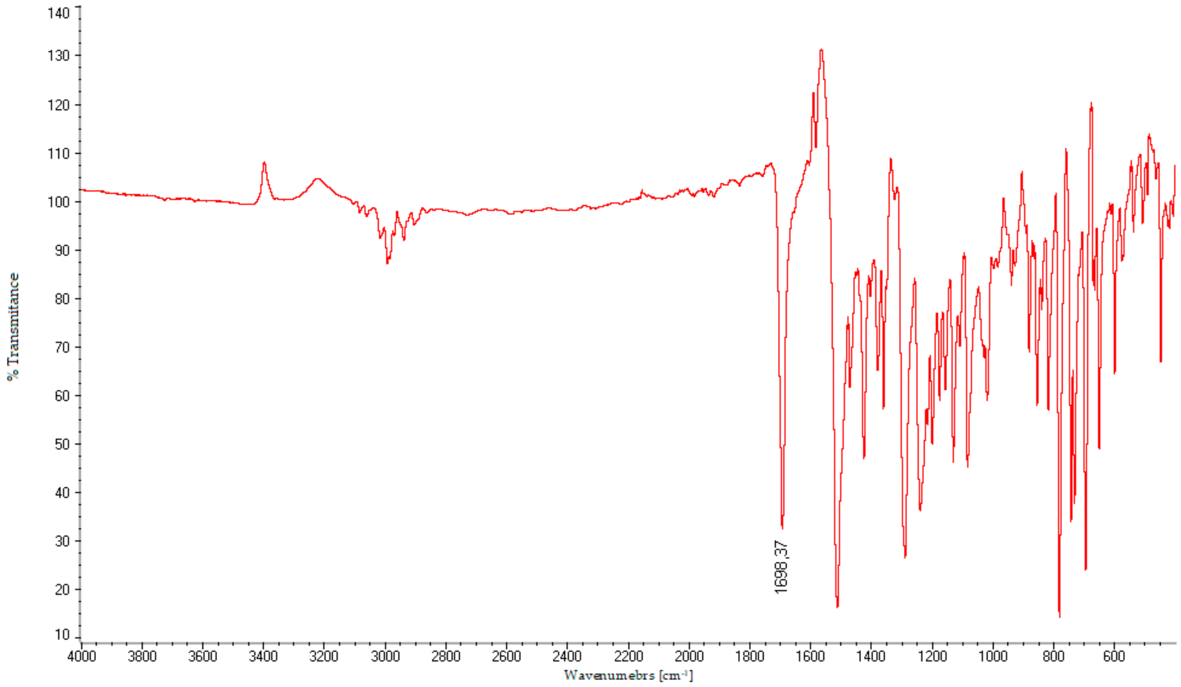

Figure S50. IR spectrum of compound **2j**.

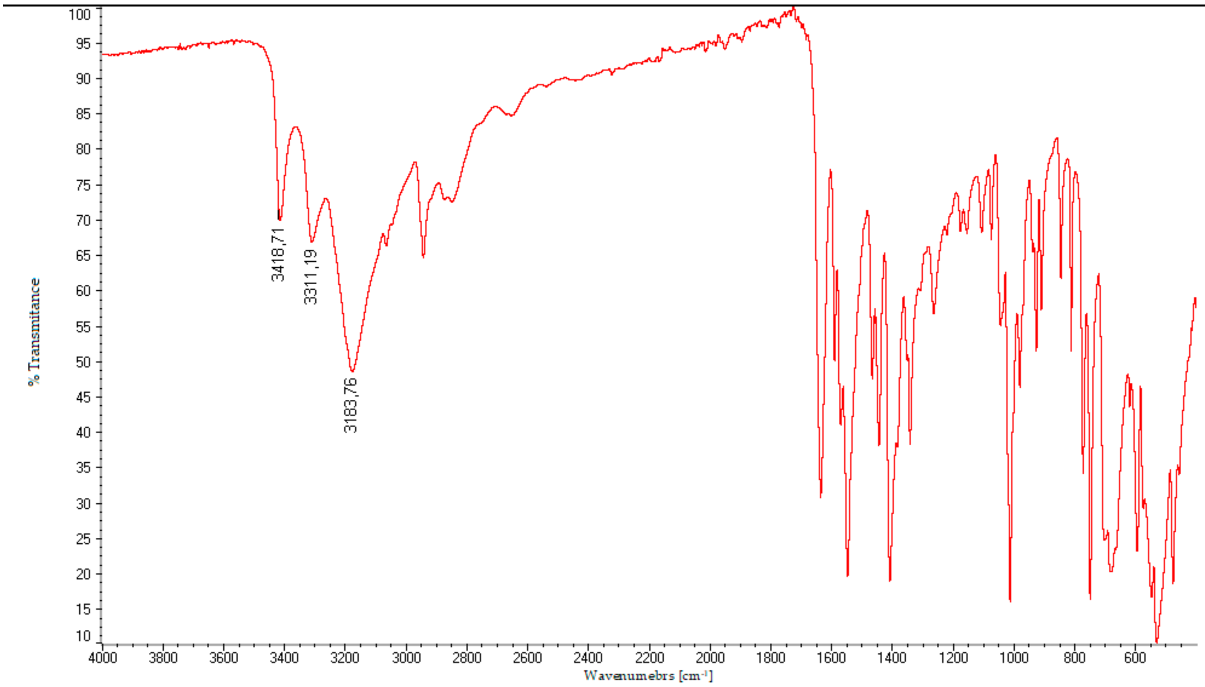

Figure S51. IR spectrum of compound **3a**.

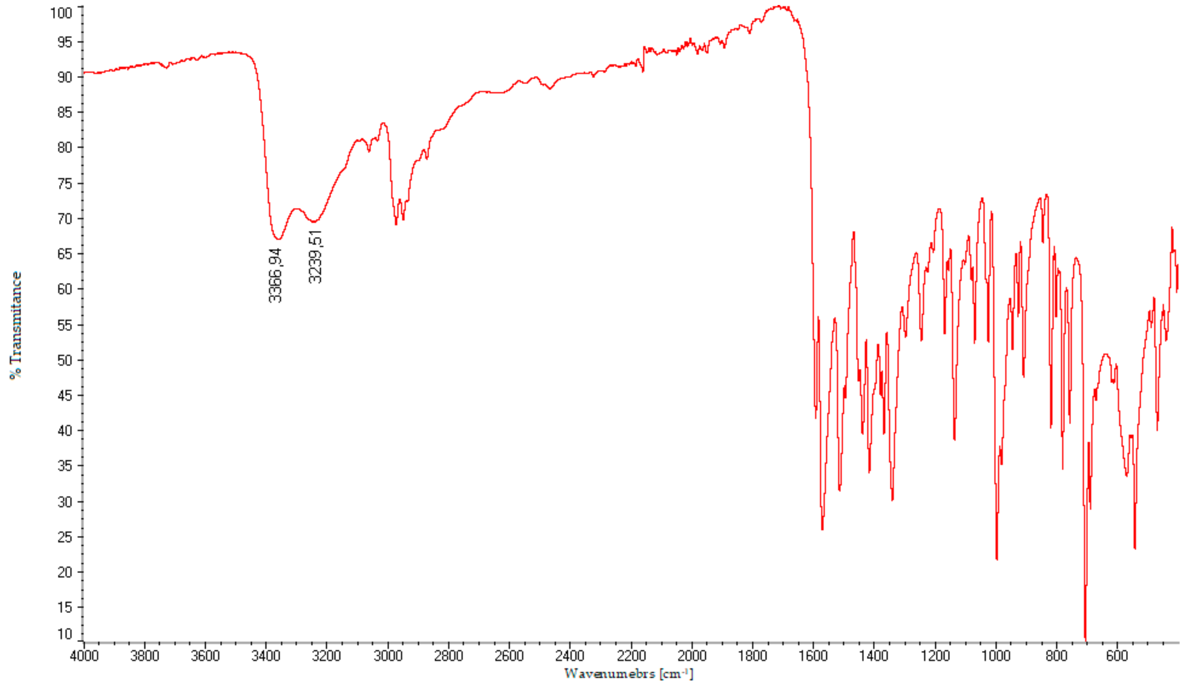

Figure S52. IR spectrum of compound **3b**.

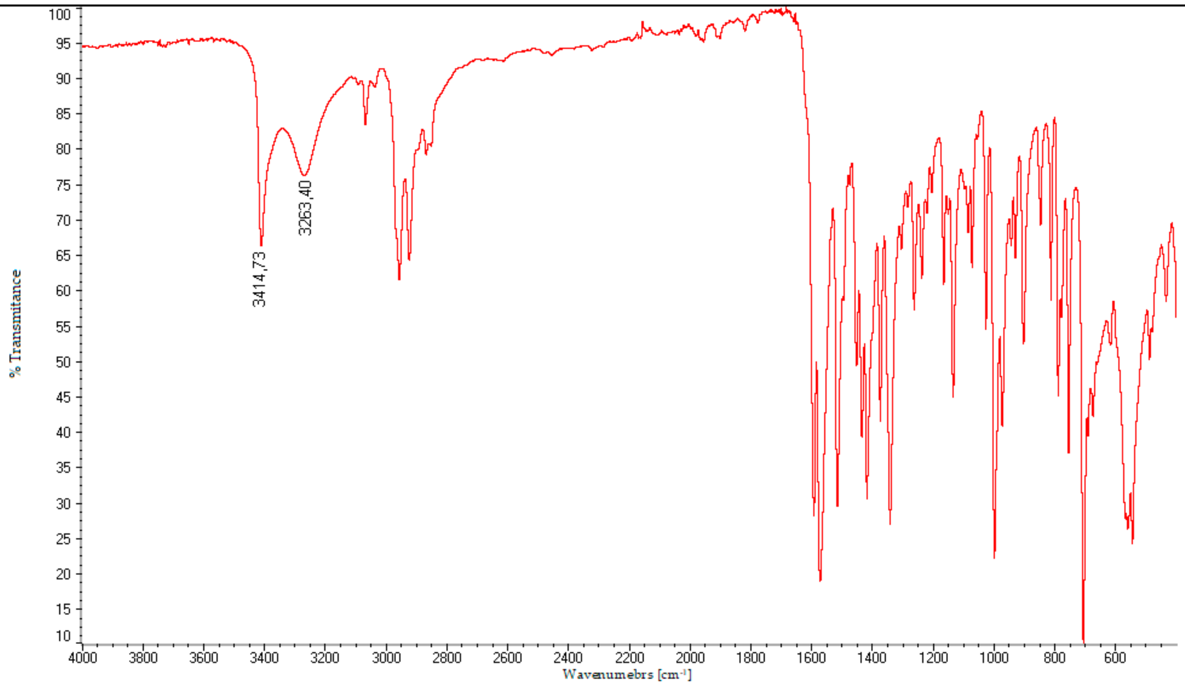

Figure S53. IR spectrum of compound **3c**.

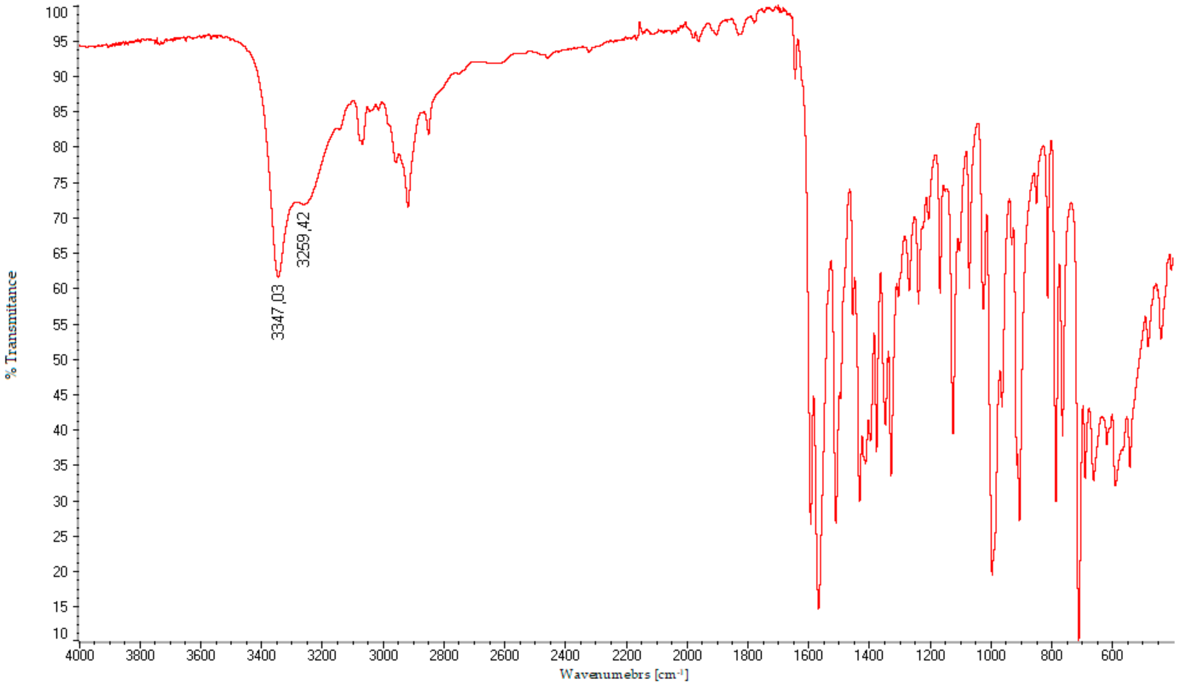

Figure S54. IR spectrum of compound **3d**.

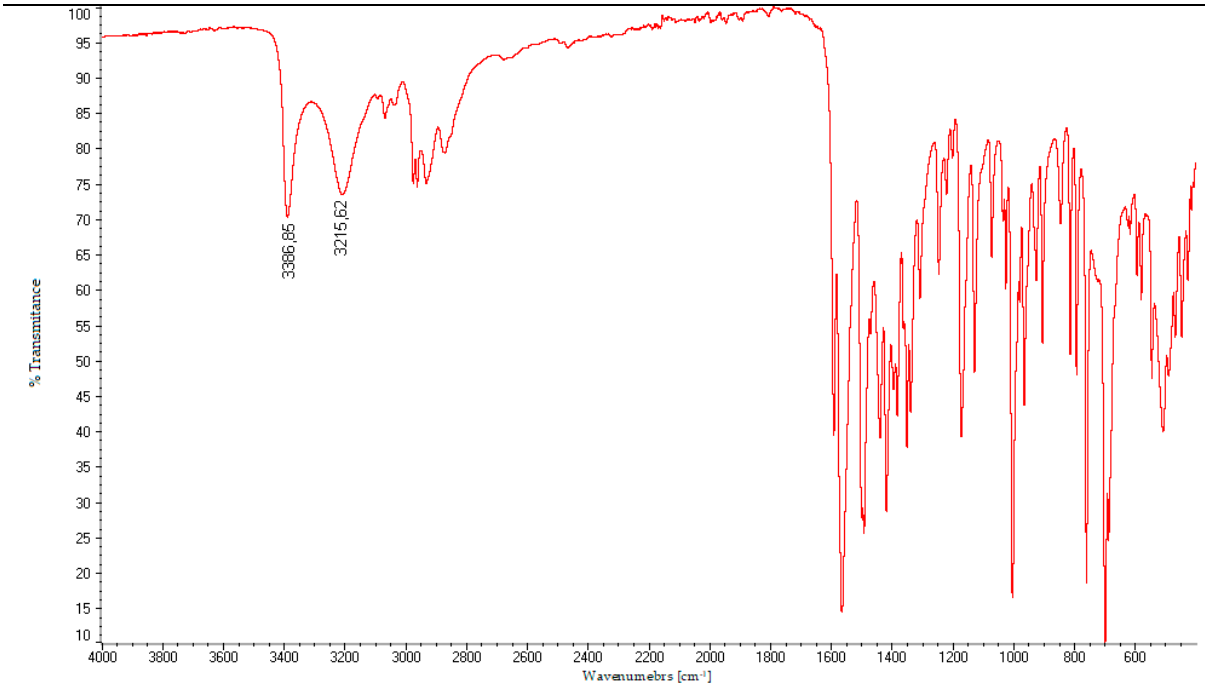

Figure S55. IR spectrum of compound **3e**.

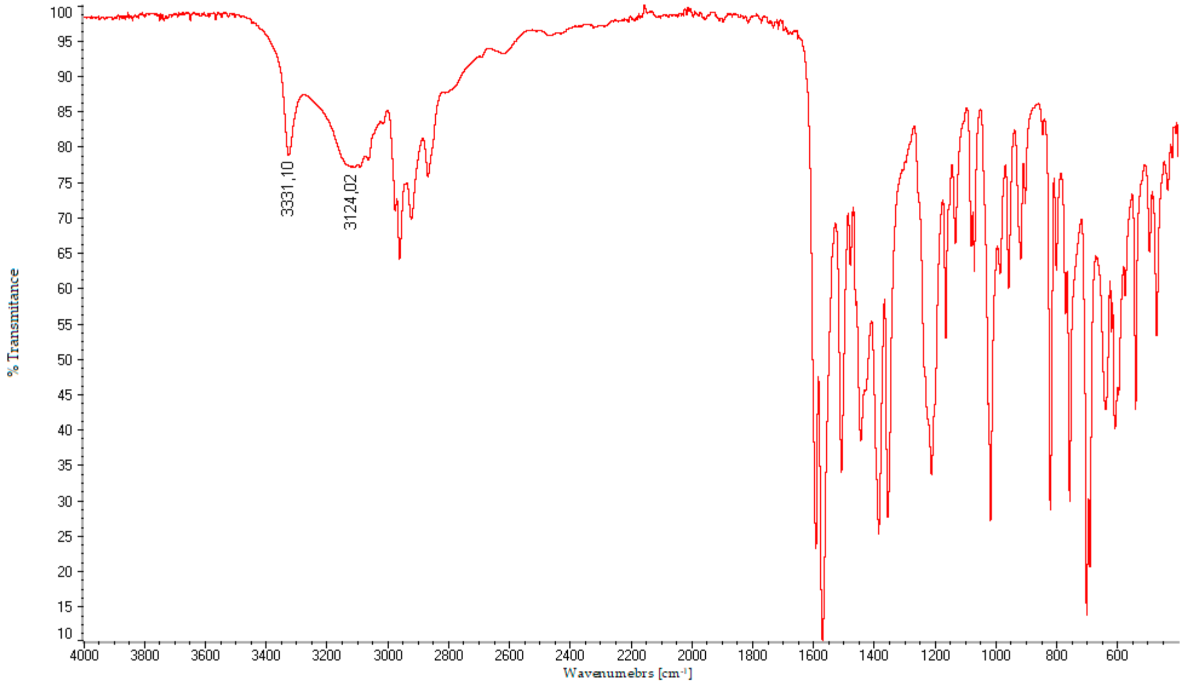

Figure S56. IR spectrum of compound **3f**.

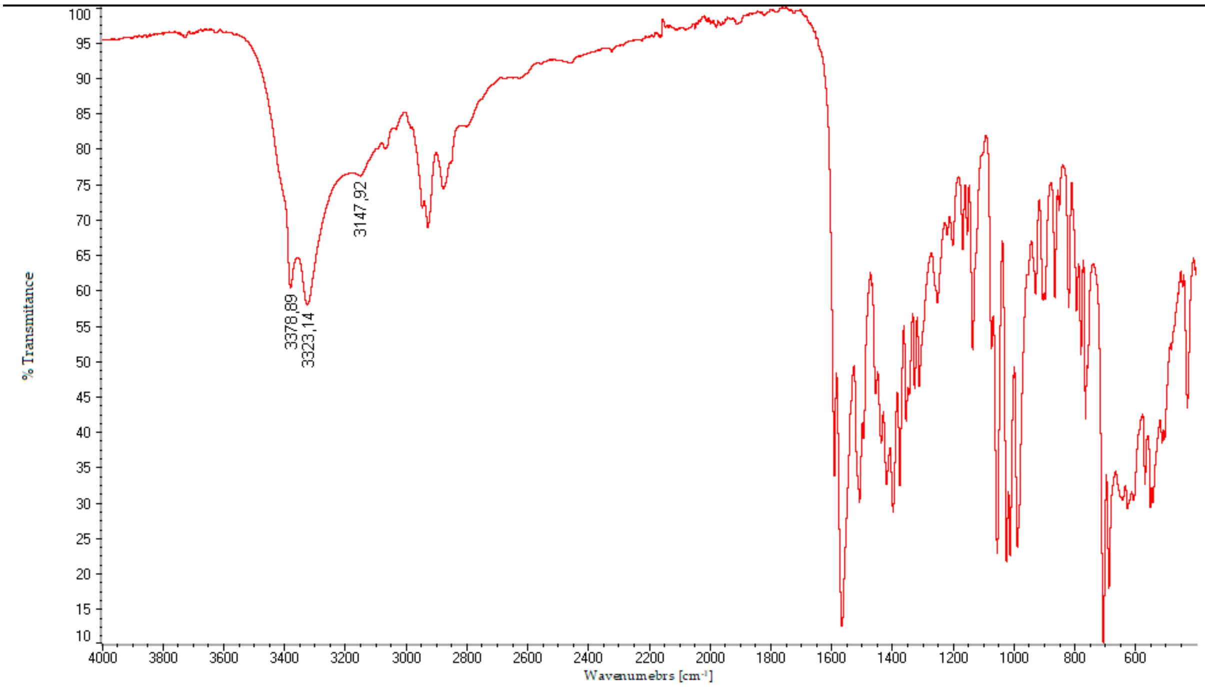

Figure S57. IR spectrum of compound **3g**.

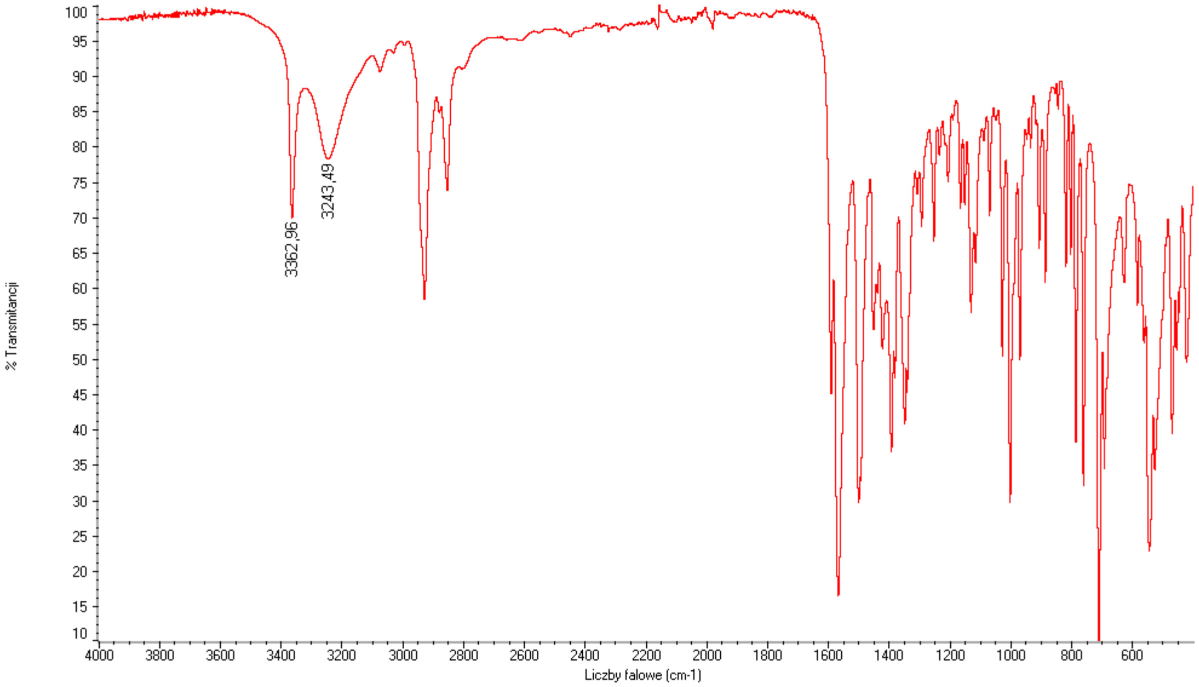

Figure S58. IR spectrum of compound **3h**.

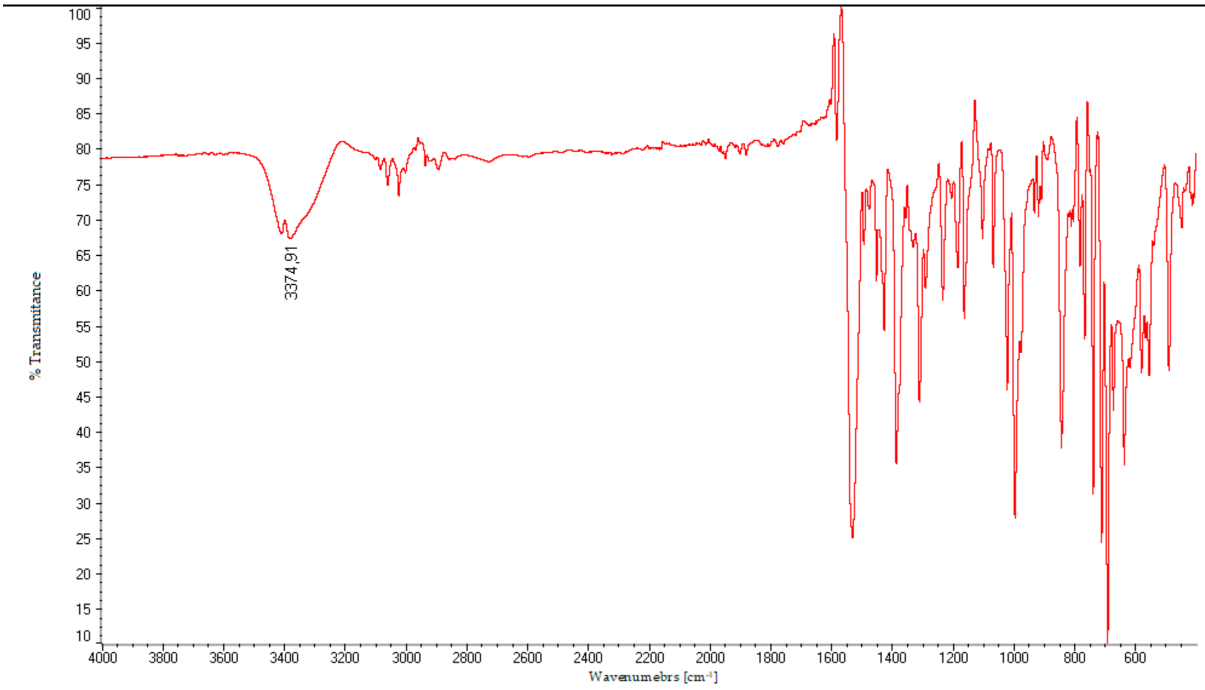

Figure S59. IR spectrum of compound **3i**.

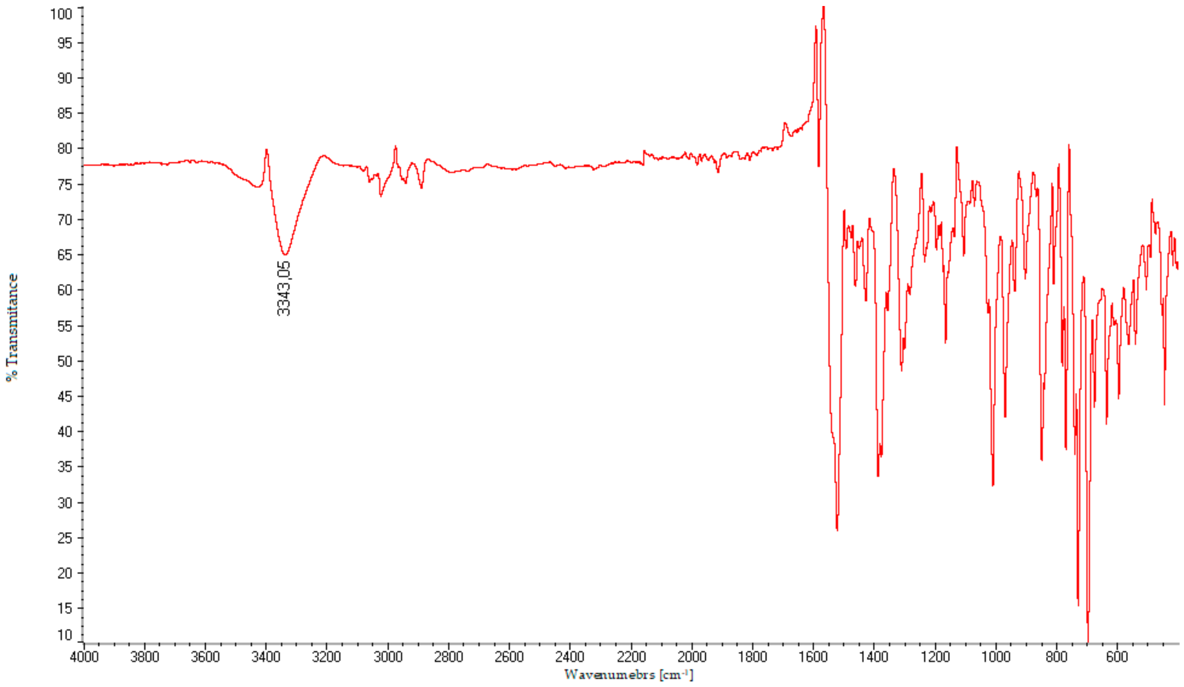

Figure S60. IR spectrum of compound **3j**.

Table S1. Selected crystallographic data and structure refinements for **3c** and **3e**-**3h**.

	**3c**	**3e**	**3f**	**3g**	**3h**
Chemical formula	$C_{12}H_{13}N_3O$	$C_{15}H_{19}N_3O$	$C_{15}H_{17}N_3O$	$C_{15}H_{19}N_3O$	$C_{16}H_{21}N_3O$
M_r (g/mol)	215.25	257.33	255.31	257.33	271.36
Crystal system, space group	monoclinic, $P2_1/c$	monoclinic, $P2_1/c$	monoclinic, $P2_1/c$	monoclinic, $P2_1/c$	orthorhombic, $Pca2_1$
Temperature (K)	200	100	100	100	100
a, *b*, *c* (Å)	8.837 (2), 13.526 (3), 9.167 (2)	7.8736 (19), 18.819 (4), 9.437 (2)	7.9441 (10), 19.061 (2), 8.8572 (10)	8.9456(16), 17.818 (4), 8.4892 (19)	9.653 (3), 27.393 (10), 11.240 (3)
α, β, γ(°)	90, 102.95 (4), 90	90, 109.14 (3), 90	90, 103.78 (2), 90	90, 101.08 (2), 90	90, 90, 90
V (Å^3)	347.46 (17)	1321.0 (6)	1302.6 (3)	1327.9 (5)	2972.1 (16)
Z	4	4	4	4	8
λ (Å)	0.71073	0.71073	1.5418	0.71073	0.71073
Crystal size (mm)	0.52 × 0.36 × 0.16	0.45 × 0.24 × 0.08	0.50 × 0.36 × 0.25	0.42 × 0.22 × 0.05	0.42 × 0.31 × 0.11
D_{calc} (g/cm^3)	1.339	1.294	1.302	1.287	1.213
μ (mm^{-1})	0.09	0.08	0.67	0.08	0.08
F (000)	456	552	544	552	1168
2θ range (°)	3.3–28.8	2.7–30.8	5.6–68.4	3.2–28.5	2.9–28.8
No of measured, independent and observed [I > 2σ(I)] reflections	5853, 2490, 1894	8814, 3737, 3082	6399, 2383, 2212	18751, 3362, 2928	14870, 6343, 4519
R_{int}	0.042	0.024	0.040	0.028	0.068
Parameters, restraints	147, 0	175, 0	174, 0	176, 0	552, 154
Goof on F^2	1.06	1.04	1.02	1.07	1.03
R_1 [I > 2σ(I)]	0.066	0.042	0.056	0.039	0.095
wR_2 (all data)	0.196	0.109	0.153	0.108	0.285
T_{min}, T_{max}	-	-	0.782, 0.876	-	-
Extinction coefficient	-	-	-	-	0.018 (5)
$\Delta\rho_{max}$, $\Delta\rho_{min}$(e Å^{-3})	0.31, −0.28	0.40, −0.22	0.34, −0.37	0.40, −0.22	0.55, −0.44
CCDC No	2102842	2102843	2102844	2102845	2102846

Table S2. Comparison of selected geometrical parameters of compounds **3c** and **3e-3h**.

	3c	3e	3f	3g	3h
Bond lengths (Å)					molecules A/B/C
O1—C51	1.425 (2)	1.4338 (13)	1.4405 (18)	1.4318 (13)	1.425 (5)/1.418 (14)/1.426 (14)
N1—C2	1.340 (2)	1.3391 (14)	1.3419 (18)	1.3424 (13)	1.341 (6)/1.319 (14)/1.331 (13)
N1—C6	1.366 (2)	1.3646 (13)	1.3600 (18)	1.3663 (13)	1.370 (5)/1.380 (14)/1.357 (14)
N3—C2	1.339 (2)	1.3356 (13)	1.3322 (18)	1.3348 (13)	1.339 (6)/1.342 (14)/1.339 (14)
N3—C4	1.344 (2)	1.3462 (13)	1.3454 (18)	1.3439 (13)	1.345 (5)/1.342 (14)/1.358 (13)
N4—C4	1.350 (2)	1.3449 (13)	1.3491 (18)	1.3514 (13)	1.355 (6)/ 1.342 (14)/1.360 (18)
N4—C41	-	1.4557 (14)	1.4569 (18)	1.4673 (13)	1.467 (6)/ 1.502 (14)/1.488 (17)
C2—C21	1.483 (3)	1.4906 (14)	1.4925 (18)	1.4924 (14)	1.480 (6)/1.484 (13)/1.474 (13)
C4—C5	1.421 (2)	1.4275 (15)	1.423 (2)	1.4246 (14)	1.434 (6)/1.429 (14)/1.412 (15)
C5—C6	1.379 (3)	1.3806 (14)	1.382 (2)	1.3863 (15)	1.377 (6)/ 1.366 (16)/1.365 (15)
C5—C51	1.503 (2)	1.5066 (15)	1.5053 (18)	1.5093 (14)	1.502 (5)/1.535 (14)/1.524 (14)
C6—C61	1.503 (3)	1.5045 (15)	1.5046 (19)	1.5084 (14)	1.501 (6)/1.515 (18)/1.516 (19)
C41—C42	-	1.5200 (18)	1.499 (2)	1.5253 (16)	1.509 (7)/1.50 (2)/1.512 (17)
Bond angles (°)					
O1—C51—C5	112.28 (15)	112.42 (9)	112.17 (11)	112.88 (8)	114.5 (3)/ 113.8 (10)/ 115.6 (10)
N1—C2—N3	126.13 (16)	126.78 (9)	126.60 (12)	126.23 (9)	125.5 (4)/ 127.2 (11)/ 126.5 (11)
N3—C4—N4	117.66 (16)	117.68 (10)	117.35 (13)	116.34 (9)	119.3 (4)/ 119.6 (11)/ 117.4 (13)
N3—C4—C5	122.24 (17)	121.27 (9)	121.48 (12)	121.38 (9)	121.3 (4)/120.8 (11)/ 121.1 (11)
N4—C4—C5	120.10 (16)	121.04 (9)	121.18 (12)	122.28 (9)	119.3 (4)/119.6 (11)/ 121.3 (13)
N4—C41—C42	-	113.33 (9)	111.01 (12)	110.98 (9)	111.9 (4)/ 106.5 (12)/ 110.2 (15)
C2—N1—C6	116.93 (15)	115.99 (9)	116.20 (12)	115.94 (9)	117.3 (4)/117.3 (12)/ 116.6 (12)
C2—N3—C4	116.51 (15)	116.95 (9)	116.92 (12)	117.59 (9)	117.5 (4)/116.1 (10)/ 116.2 (10)
Torsion angles (°)					
O1—C51—C5—C4	−53.9 (2)	63.31 (12)	64.47 (17)	−62.94 (12)	49.0 (6)/−50.0 (19)/46 (2)
O1—C51—C5—C6	125.68 (19)	−116.12 (11)	−112.34 (15)	117.49 (11)	−133.5 (5)/136.8 (17)/−137.6 (18)
N1—C2—N3—C4	2.6 (3)	−1.31 (15)	0.9 (2)	2.80 (15)	0.1 (7)/1 (2)/−2 (2)
N1—C2—C21—C22	−25.2 (2)	−10.80 (15)	−12.00 (19)	−15.50 (14)	−22.9 (7)/16 (2)/−15 (2)

N1—C2—C21—C26	158.12 (17)	169.55 (9)	168.84 (13)	167.84 (9)	159.6 (5)/−161.7 (19)/160.0 (16)
N3—C2—N1—C6	−2.3 (3)	−2.50 (15)	−3.3 (2)	−3.78 (14)	−1.8 (7)/3 (3)/−3 (2)
N3—C4—C5—C6	−1.7 (2)	−2.02 (15)	−2.1 (2)	−2.19 (14)	−1.3 (7)/−3 (2)/0 (3)
N3—C2—C21—C22	151.94 (18)	169.71 (9)	168.15 (12)	163.56 (9)	158.2 (5)/ −161.5 (15)/166.3 (15)
N3—C2—C21—C26	−24.7 (2)	−9.93 (14)	−11.00 (19)	−13.11 (13)	−19.3 (7)/21 (2)/ −19 (2)
N3—C4—N4—C41	-	−6.83 (15)	17.1 (2)	0.42 (14)	10.7 (7)/−12 (3)/11 (4)
N4—C4—N3—C2	179.89 (16)	−177.36 (9)	−178.39 (12)	−179.33 (9)	−180.0 (4)/−180.0 (15)/178 (2)
N4—C4—C5—C6	177.95 (16)	178.95 (9)	178.24 (13)	177.52 (9)	−179.8 (5)/175.8 (17)/−175 (2)
N4—C4—C5—C51	−2.4 (2)	−0.52 (15)	1.2 (2)	−2.09 (14)	−2.2 (7)/2 (2)/ 1 (3)
C2—N1—C6—C61	−179.07 (15)	−174.33 (9)	−176.38 (11)	−178.88 (8)	−177.0 (4)/−176.6 (17)/−176 (2)
C4—N4—C41—C42	-	105.57 (12)	79.14 (17)	77.54 (12)	−63.0 (7)/63 (3)/−66 (3)
C5—C4—N4—C41	-	172.23 (10)	−163.30 (13)	−179.31 (9)	−170.7 (4)/ 168.5 (15)/−173 (2)
C51—C5—C6—C61	1.2 (3)	−4.23 (16)	−4.2 (2)	1.25 (15)	0.7 (8)/−11 (3)/1 (3)

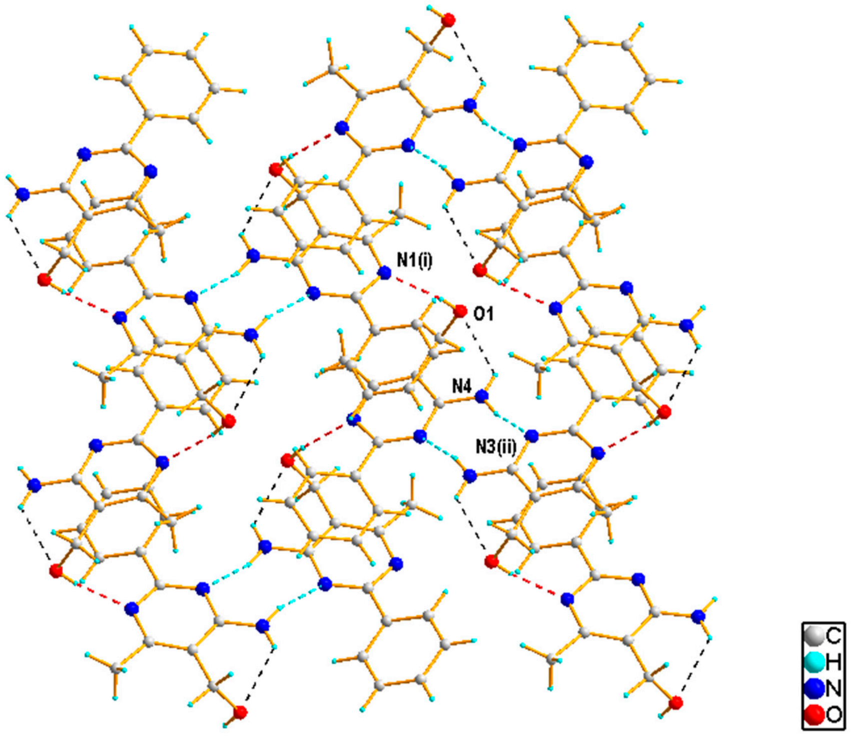

Figure S61. A packing diagram of **3c** viewed along the a-axis, showing the formation of 2D hydrogen-bonded network through intermolecular interactions, with O—H···N in red and N—H···N in cyan. Dashed lines in black indicate intramolecular N—H···O hydrogen bonds. Symmetry codes: (i).x, $-y$+3/2, z−1/2 and (ii) $-x$+1, $-y$+1, $-z$.
